# DNA methylation underpins the epigenomic landscape regulating genome transcription in *Arabidopsis*

**DOI:** 10.1186/s13059-022-02768-x

**Published:** 2022-09-20

**Authors:** Lun Zhao, Qiangwei Zhou, Li He, Li Deng, Rosa Lozano-Duran, Guoliang Li, Jian-Kang Zhu

**Affiliations:** 1 National Key Laboratory of Crop Genetic Improvement, National Engineering Research Center of Rapeseed, Hubei Hongshan Laboratory, Huazhong Agricultural University, Wuhan, 430070 China; 2 Shanghai Center for Plant Stress Biology, Center for Excellence in Molecular Plant Sciences, Chinese Academy of Sciences, Shanghai, 201602 China; 3 Department of Plant Biochemistry, Centre for Plant Molecular Biology (ZMBP), Eberhard Karls University, D-72076 Tübingen, Germany; 4 Hubei Key Laboratory of Agricultural Bioinformatics and Hubei Engineering Technology Research Center of Agricultural Big Data, Huazhong Agricultural University, Wuhan, 430070 China; 5 Institute of Advanced Biotechnology and School of Life Sciences, Southern University of Science and Technology, Shenzhen, 518055 China; 6 Center for Advanced Bioindustry Technologies, Chinese Academy of Agricultural Sciences, Beijing, 100081 China

**Keywords:** DNA methylation-free plant, Histone modification, Chromatin state

## Abstract

**Background:**

It is challenging to determine the effect of DNA methylation on the epigenetic landscape and the function in higher organisms due to the lack of DNA methylation-free mutants.

**Results:**

Here, the analysis of a recently generated *Arabidopsis* mutant completely devoid of DNA methylation reveals that DNA methylation underpins the genome-wide landscape of histone modifications. Complete loss of DNA methylation causes an upheaval of the histone modification landscape, including complete loss of H3K9me2 and widespread redistribution of active and H3K27me3 histone marks, mostly owing to the role of DNA methylation in initiating H3K9me2 deposition and excluding active marks and repressive mark H3K27me3; CG and non-CG methylation can act independently at some genomic regions while they act cooperatively at many other regions. The transcriptional reprogramming upon loss of all DNA methylation correlates with the extensive redistribution or switches of the examined histone modifications. Histone modifications retained or gained in the DNA methylation-free mutant serve as DNA methylation-independent transcriptional regulatory signals: active marks promote genome transcription, whereas the repressive mark H3K27me3 compensates for the lack of DNA hypermethylation/H3K9me2 at multiple transposon families.

**Conclusions:**

Our results show that an intact DNA methylome constitutes the scaffolding of the epigenomic landscape in *Arabidopsis* and is critical for controlled genome transcription and ultimately for proper growth and development.

**Supplementary Information:**

The online version contains supplementary material available at 10.1186/s13059-022-02768-x.

## Background

DNA methylation (5-methylcytosine) is crucial for the control of transposon activity and gene expression [1–5]. DNA methylation in plants occurs in three sequence contexts: CG, CHG, and CHH (H = A, T, or C) [6]. CG methylation is catalyzed by MET1 (ortholog of mammalian DNMT1) [1, 6, 7], while four DNA methyltransferases are collectively responsible for non-CG methylation [8]: the chromomethylases CMT2 and CMT3 for CHG [1, 9] and DRM1/2 (orthologs of mammalian DNMT3) and CMT2 for CHH [8, 9]. De novo DNA methylation is mediated by DRM2 through the RNA-directed DNA methylation pathway [3, 6, 10, 11]. Methylation of CG and non-CG occurs interdependently in many contexts [9, 12]. Both CG methylation and non-CG methylation are present in transposable elements (TEs), whereas CG-only methylation is prevalent in gene bodies [6, 13].

DNA methylation and histone modifications constitute the epigenomic landscape that determines transcriptional activities [14, 15]. The availability of some DNA methylation-related mutants has paved the way for our understanding of the interplay between DNA methylation and histone modifications in *Arabidopsis* [16]. Disruption of MET1 results in loss of CG methylation throughout the genome [9] and causes ectopic gain of H3K9me2 (histone H3 lysine 9 dimethylation) and H3K27me3 (histone H3 lysine 27 trimethylation) [17]. In *drm1 drm2 cmt2 cmt3* (*ddcc*) quadruple mutant plants, in which non-CG methylation is completely lost, H3K9me2 was reportedly strongly reduced [8]. Non-CG methylation and H3K9me2 reinforce each other through a feedback loop: methylated CHG and CHH recruit the H3K9-specific methyltransferases SUVH4, SUVH5, and SUVH6, and in turn, H3K9me2 facilitates CMT3 and CMT2 function to methylate DNA in the CHG and/or CHH context [8, 18–20]. The histone acetyltransferase IDM1 is required for the DNA demethylase ROS1 to remove DNA methylation in some genomic regions [3, 21]; histone deacetylase 6 (HDA6) interacts with MET1, which methylates DNA [22, 23]; alterations in other histone marks (including H3K36me3, H3K27me1, H3K4me3, and H3K27me3) do not have a notable impact on DNA methylation [9]. Moreover, DNA methylation has a profound impact on chromatin accessibility [24, 25]. However, the inability to generate plant materials completely devoid of DNA methylation [26, 27] has precluded a comprehensive analysis of the extent of contribution of DNA methylation to chromatin states, and how these states ultimately influence gene expression.

Here, by integrating genome-wide DNA methylation and histone modification marks, as well as transcriptional profiling in wild type (Col-0), *met1*, *ddcc*, and the quintuple mutant *mddcc* (*met1 drm1 drm2 cmt2 cmt3*) *Arabidopsis* plants, we determined the impact of complete loss of DNA methylation on the histone modification landscape and the relative contributions of CG and non-CG methylation to this landscape. We found that DNA methylation negatively affects the presence of active marks as well as the repressive mark H3K27me3 but is an essential precondition for H3K9me2 deposition. Importantly, our results suggested critical roles of different histone modifications in genome-wide transcriptional regulation in the absence of DNA methylation, unveiling that active marks promote genome transcription, whereas the repressive mark H3K27me3 compensates for the lack of DNA hypermethylation/H3K9me2 at multiple transposon families throughout the genome. Together, our results provide comprehensive insights into the crucial contribution of DNA methylation in shaping the chromatin states to ensure appropriate transcriptional control in plants.

## Results

### Epigenome and transcriptome profiling of DNA methylation-free *Arabidopsis*

**Fig. 1:**
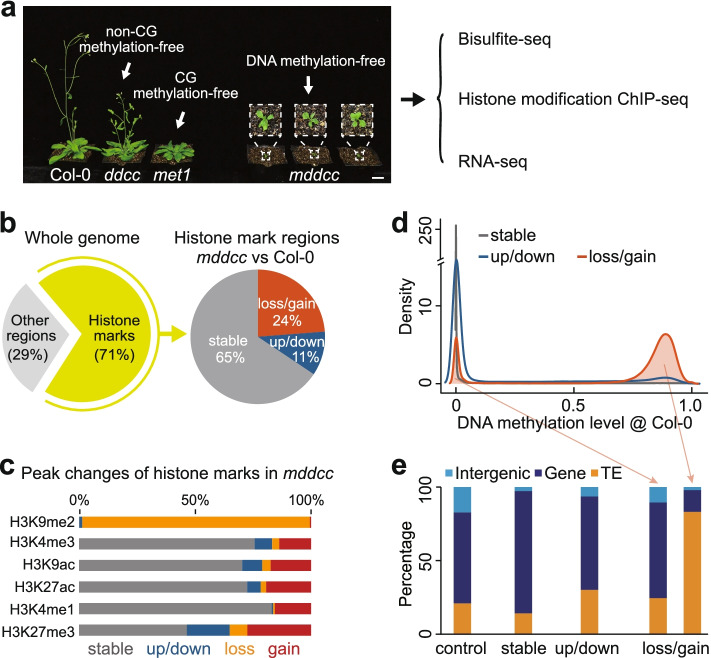
Complete loss of DNA methylation causes upheaval of the histone modification landscape. **a** Wild type (Col-0), *ddcc* (*drm1 drm2 cmt2 cmt3*), *met1*, and *mddcc* (*met1 drm1 drm2 cmt2 cmt3*) mutant plants and data generation in this study. Five-week-old plants are shown. Bar, 2 cm. **b** Changes of histone modifications in *mddcc* compared to the wild type. Six representative histone marks (H3K4me3, H3K9ac, H3K27ac, H3K4me1, H3K27me3, and H3K9me2) were investigated. These marks occupied 71% of the wild type and/or *mddcc* genome (left panel) and were divided into three categories based on their behavior in the mutant: loss/gain, up/downregulated, and stable/unchanged (right panel). **c** Peak changes of the indicated histone marks in *mddcc* compared with the wild type. **d** Distribution of DNA (CG) methylation levels in the wild type for the three categories of histone modification regions in (**b**). **e** Genomic distribution of the indicated categories in (**d**). The loss/gain category was divided into two sub-categories based on DNA methylation levels

To completely eliminate DNA methylation, we recently generated the quintuple mutant *mddcc* (*met1 drm1 drm2 cmt2 cmt3*), in which all reported DNA methyltransferases were mutated [28]. As expected, almost all CG and non-CG methylation is eliminated in *met1* and *ddcc*, respectively, revealed by examinations of both two- [28] and five-week-old mutant plants (Additional file 1: Fig. S1) used in this study; DNA methylation in all contexts is completely eliminated in *mddcc* mutant, which grew extremely slowly and failed to flower [28] (Fig. 1a).

We employed enhanced ChIP-seq (eChIP-seq) [29] and RNA-seq to generate the genome-wide profiles of six representative histone modifications (H3K4me3, H3K9ac, H3K27ac, H3K4me1, H3K27me3, and H3K9me2), RNA Polymerase II (RNAPII), and transcriptomes in wild type (Col-0), *ddcc*, *met1*, and *mddcc* plants (Additional file 2: Table S1). H3K4me3, H3K9ac, and H3K27ac are associated with active promoter regions; H3K4me1 is associated with transcribed regions; H3K27me3 is deposited by Polycomb group proteins and is associated with facultative heterochromatin; and H3K9me2 is associated with constitutive heterochromatin [29–31]. Experiments were performed using five-week-old *mddcc* plants (2-week-old *mddcc* plants were too small to collect enough materials for the experiments) and both 2-week-old (more similar to 5-week-old *mddcc*) and 5-week-old plants for Col-0, *ddcc*, and *met1*. Collectively, 140 high-quality datasets were generated (Additional file 2: Table S1). Given the similar histone modification patterns observed in two- and five-week-old plants (Additional file 1: Fig. S2), we used the former for subsequent analyses.

### Upheaval of the histone modification landscape in the *mddcc* mutant

A total of ~71% of the *Arabidopsis* genome was modified by histone marks in both the wild type and *mddcc* (Fig. 1b). Detailed analyses revealed that ~35% of the histone-modified regions was variable in *mddcc*, either through loss/gain (24%) or through up/downregulation (11%) of histone modifications (Fig. 1b). The loss/gain category involved all types of histone marks, including complete loss of the heterochromatin mark H3K9me2 in *mddcc* (see below), whereas the up/downregulated and stable (unchanged) categories mainly involved active marks and the repressive mark H3K27me3 (Fig. 1c). Accordingly, the former category exhibited different genomic properties from the latter two categories. Loss/gained histone marks were mainly located in heavily DNA methylated genomic regions in the wild type background, preferentially targeted transposable elements (TEs), and were enriched in pericentromeric heterochromatin (Fig. 1d, e, and Additional file 1: Fig. S3). In contrast, stable/unchanged histone marks, as well as those up-/down-regulated marks, were mostly distributed in low methylation genomic regions and gene regions (including promoters) (Fig. 1d, e). Collectively, our data indicate that DNA methylation has a profound effect on the histone modification landscape.

### Impact of DNA methylation on H3K9me2

**Fig. 2:**
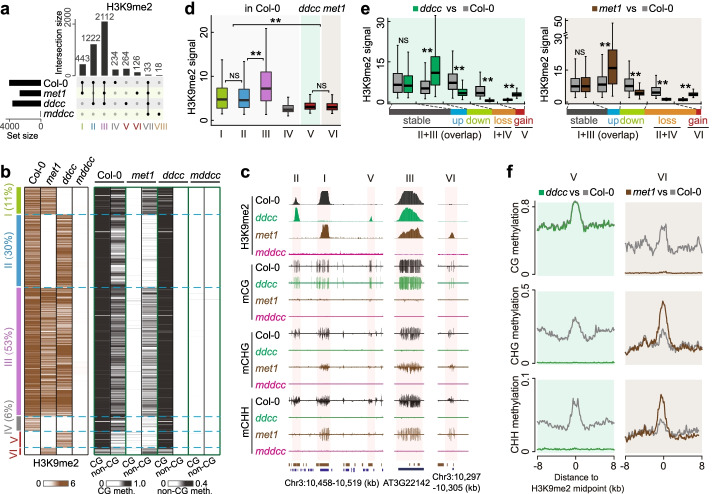
Dissecting the relative contributions of CG and non-CG methylation to the H3K9me2 pattern. **a** Upset plot displaying the number of unique and overlapping H3K9me2 loci between the wild type and indicated mutants. Bars represent the intersection size for overlapping H3K9me2 loci between Col-0, *met1*, *ddcc*, and *mddcc*. Numbers (> 10) of H3K9me2 loci in different subgroups are shown. All H3K9me2 loci were classified into eight types as indicated. **b** Heatmaps of H3K9me2 levels and DNA methylation enrichments of the indicated H3K9me2 types in (**a**). The number of H3K9me2 loci in the wild type is defined as 100%, and the percentages of type I–IV are shown. **c** Representative examples of H3K9me2 changes caused by DNA methylation loss. **d** Peak intensities of the indicated H3K9me2 types in different genotypes. **e** H3K9me2 intensities of the indicated categories in *ddcc* (left) and *met1* (right) compared with the wild type. The horizontal column at the bottom shows the proportion of H3K9me2 in different categories. Boxplots in **d** and **e** include a median with quartiles and outliers above the top whisker. The statistical analysis was performed using two-side Wilcoxon test. NS, no significant difference. **p < 2.2e−16 from Wilcoxon test. **f** Distribution of DNA methylation at the H3K9me2 loci gained in *ddcc* (type V) and *met1* (type VI) compared with the wild type

We explored the effect of DNA methylation on H3K9me2. First, we evaluated the impact of total DNA methylation on the genome-wide distribution of H3K9me2. Compared to more than 4000 H3K9me2 loci called in the wild type (Additional file 1: Fig. S4; see Methods), only a few dozen H3K9me2 loci were identified in *mddcc* (Fig. 2a, type VII and VIII). With careful scrutiny, we found that the H3K9me2 signals in *mddcc* were noise based on the genome browser views (Additional file 1: Fig. S5, S6): similar signals were observed in both the H3K9me2 ChIP and input (control), and their peak intensities were extremely low. Consistent with this notion, high transcript levels were detected at these H3K9me2 loci in *mddcc*, indicating that these loci are active genomic regions rather than inactive/heterochromatic regions. All 15 annotated H3K9 methyltransferase genes (*SUVH1–10* and *SUVR1–5*) [32–34] had similar transcript levels and epigenetic patterns in *mddcc* and wild-type plants (Additional file 1: Fig. S7), ruling out the possibility that differences in the expression of these enzymes caused the observed effects on genome-wide H3K9me2 distribution. Thus, we concluded that complete loss of DNA methylation results in full loss of H3K9me2 genome-wide. H3K9me2 is preferentially located in heavily DNA methylated genomic regions in the wild type [30, 32] (Fig. 2b, c; Additional file 1: Fig. S8a, S8b), supporting that heavy DNA methylation is a pre-requisite for H3K9me2 deposition.

Next, we dissected the relative contribution of CG and non-CG methylation to the distribution of H3K9me2 using *ddcc*, *met1*, and *mddcc* mutants. Compared with the wild type, ~11% (type I) and ~30% (type II) of H3K9me2 loci were lost specifically in *ddcc* and *met1*, respectively; only ~6% (type IV) were lost in both *ddcc* and *met1* (Fig. 2a–c; Additional file 1: Fig. S8a). Considering the interdependence of CG and non-CG methylation [9], we confirmed that most of the Type IV H3K9me2 loci were controlled by either CG or non-CG methylation based on the combined patterns of CG and non-CG methylation changes in *ddcc* and *met1* mutants (Additional file 1: Fig. S8a, S8c). These results indicate that CG methylation has a greater effect on H3K9me2 at these loci than non-CG methylation. In contrast, ~53% (type III) of H3K9me2 loci were lost in *mddcc* specifically, but were retained in both *ddcc* and *met1* (Fig. 2a–c). Type III H3K9me2 loci exhibited the highest H3K9me2 abundance in the wild type (Fig. 2d) and were preferentially enriched in Gypsy retrotransposons (Additional file 1: Fig. S8d). Collectively, our data indicate that CG and non-CG methylation independently regulate H3K9me2 distribution at some loci while they cooperatively regulate H3K9me2 distribution at many more loci.

Both CG methylation and non-CG methylation have a large impact on the abundance of H3K9me2 retained in *ddcc* and *met1* mutants. Compared with the wild type, approximately ~28% and ~30% of up/downregulated H3K9me2 loci were observed in *ddcc* and *met1*, respectively (Fig. 2a, e). These H3K9me2 loci include both upregulated and downregulated regions (Fig. 2e), suggesting that DNA methylation can impact H3K9me2 through different mechanisms.

Interestingly, we found that some genomic regions gained ectopic H3K9me2 in *ddcc* and *met1* (hereafter referred to as type V and VI loci, respectively) (Fig. 2a–c). Both type V and VI loci exhibited lower H3K9me2 levels compared with type I–III loci (Fig. 2c–e). There was no significant difference in the CG methylation levels of Type V H3K9me2 loci between *ddcc* and the wild type, both showing high DNA methylation (Fig. 2b, c, f); Type V H3K9me2 loci may arise as a result of the strong downregulation of the H3K9 demethylase *IBM1* [35] in *ddcc* (Additional file 1: Fig. S9). Type VI H3K9me2 loci, gained in *met1*, displayed increased levels of both CHG and CHH methylation, similar to those at the loci displaying H3K9me2 in the wild type, at individual loci as well as in a genome-wide manner (Fig. 2b, c, f; Additional file 1: Fig. S8a, S8d). This result indicates that high non-CG methylation is required for the deposition of H3K9me2 and the feedforward loop between non-CG methylation and H3K9me2 [17, 32] is active at type VI loci in a CG methylation-independent manner.

Overall, these results demonstrate that heavy DNA methylation plays an important role in determining the genome-wide distribution of H3K9me2, which can be independently, redundantly, or interdependently regulated by CG and non-CG methylation. At least four mechanisms function to establish H3K9me2, depending on CG methylation only, non-CG methylation only, CG or non-CG methylation, and both CG and non-CG methylation; therefore, the interplay between DNA methylation and H3K9me2 goes beyond the well-known feedback regulatory loop between non-CG methylation and H3K9me2.

### Heavy DNA methylation repels active marks, H3K27me3, and H3K4me1

**Fig. 3:**
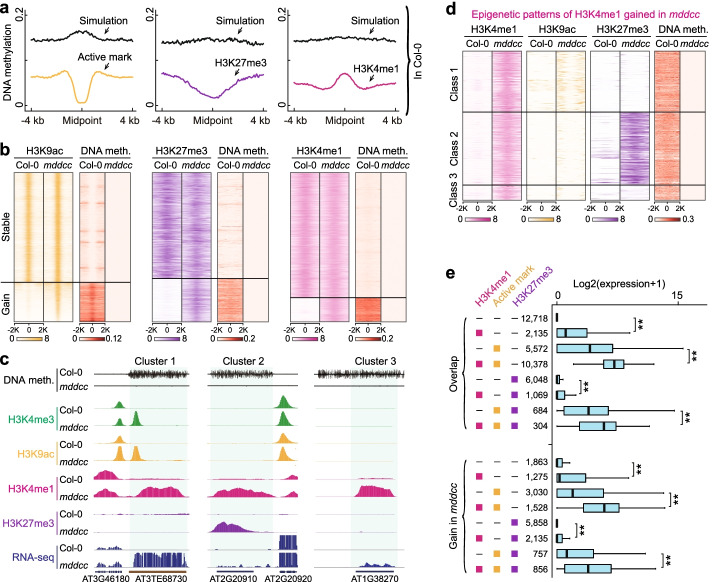
High levels of DNA methylation repel active marks, H3K27me3, and H3K4me1. **a** Association of DNA methylation surrounding the indicated histone modifications in the wild type. In our simulation, un-modified regions were uniformly sampled as simulated peaks, and the lengths of the simulated peaks follow the similar length distribution of the real peaks. **b** Heatmap of the indicated histone modification categories and DNA methylation changes in the wild type and *mddcc*. **c**, **d** Epigenetic patterns of H3K4me1 loci gained in *mddcc* and representative examples. These H3K4me1 loci were classified into three clusters: cluster 1, H3K4me1-H3K9ac/active cluster; class 2, H3K4me1-H3K27me3 bivalent cluster; class 3, H3K4me1 only cluster. **e** Expression levels of genes/TEs marked by different histone modifications in *mddcc*. “-” indicates absence. Each histone modification combination is divided into two groups: stable between *mddcc* and the wild type and gain in *mddcc*. The numbers indicate the sample size used in the analysis. Boxplots include a median with quartiles and outliers above the top whisker. The statistical analysis was performed using two-side Wilcoxon test. **p < 2.2e−16 from Wilcoxon test

The DNA methylation-free mutant plants also displayed genome-wide gain of active marks, H3K27me3, and H3K4me1, involving ~18% of modified histone regions and 8599 loci (Fig. 1c). To investigate the relationship between DNA methylation and these histone marks, we first assessed their correlation in the wild type. As expected, we found that DNA methylation was generally excluded from regions where active marks or H3K27me3 was deposited (Fig. 3a). In contrast, regions without active marks or H3K27me3 frequently showed heavy DNA methylation (Fig. 3a). H3K4me1-modified regions were also associated with lower DNA methylation levels compared to H3K4me1-unmodified regions (simulation) (Fig. 3a). Thus, active marks, H3K27me3, and H3K4me1 show anti-correlation with heavy DNA methylation, in agreement with previous results [29, 31, 36, 37].

Furthermore, both individual loci and genome-wide analyses indicated that regions with active marks, H3K27me3, or H3K4me1 gained in *mddcc* and/or *met1* were pre-marked with a high level of DNA methylation in the wild type (Fig. 3b, c; Additional file 1: Fig. S10). Previous studies found that some genomic loci were specifically marked by H3K27me3 in the endosperm, whereas these loci were modified by DNA methylation in vegetative tissues [38]. Collectively, these results demonstrate that heavy DNA methylation prevents the deposition of active marks, H3K27me3, and H3K4me1, and reinforce the notion that a state of low or no DNA methylation is required for the establishment of these histone modifications.

Next, we focused on H3K4me1-associated chromatin states. We found that H3K4me1 was usually accompanied by H3K4me3 and/or H3K27me3 (class1 and 2), a trend that was more pronounced in regions where these marks were gained in *mddcc* (Fig. 3c, d). We used ChromHMM [39] to segment the genome of each genotype into 12 chromatin states (CS) based on the combinatorial patterns of histone marks and RNAPII occupancy (Additional file 1: Fig. S11). Notably, H3K4me1-H3K27me3 (CS8), a novel bivalent chromatin state recently discovered in *Brassica napus* [37], was widely distributed across the *Arabidopsis* genome (Additional file 1: Fig. S11). Compared with the classic bivalent state H3K4me3-H3K27me3 (CS4, covering ~1.5–1.8% of the genome), H3K4me1-H3K27me3 accounted for a larger genome proportion in the wild type (2.6%) and *mddcc* (6.1%) and was enriched in gene bodies as well as TE regions with low transcript levels (Additional file 1: Fig. S11). These results suggest that H3K4me1-H3K27me3 is a predominant bivalent state in dicotyledons, unlike that in monocotyledons such as rice [29].

To better understand the role of H3K4me1 in transcriptional regulation, we examined the expression of genes/TEs harboring different combinations of histone marks in *mddcc*. Each histone modification combination was divided into two groups: retained in *mddcc* and gained in *mddcc* (Fig. 3e). We obtained similar results in these two groups. In contrast to the genes/TEs without histone marks, which showed low or no expression, the genes/TEs marked with H3K4me1 only showed relatively high expression, which was nevertheless lower than that of genes/TEs marked with H3K4me3 only (Fig. 3e). Moreover, compared with genes marked with H3K4me3 only, H3K27me3 only, or both, the expression levels of genes marked with H3K4me1 in addition to these marks were significantly higher (Fig. 3e). Taken together, these observations suggest that H3K4me1 is an active mark, albeit weaker than H3K4me3, and that it has an effect in promoting transcription together with other active marks.

### Complete loss of DNA methylation causes extensive switches of chromatin states

**Fig. 4:**
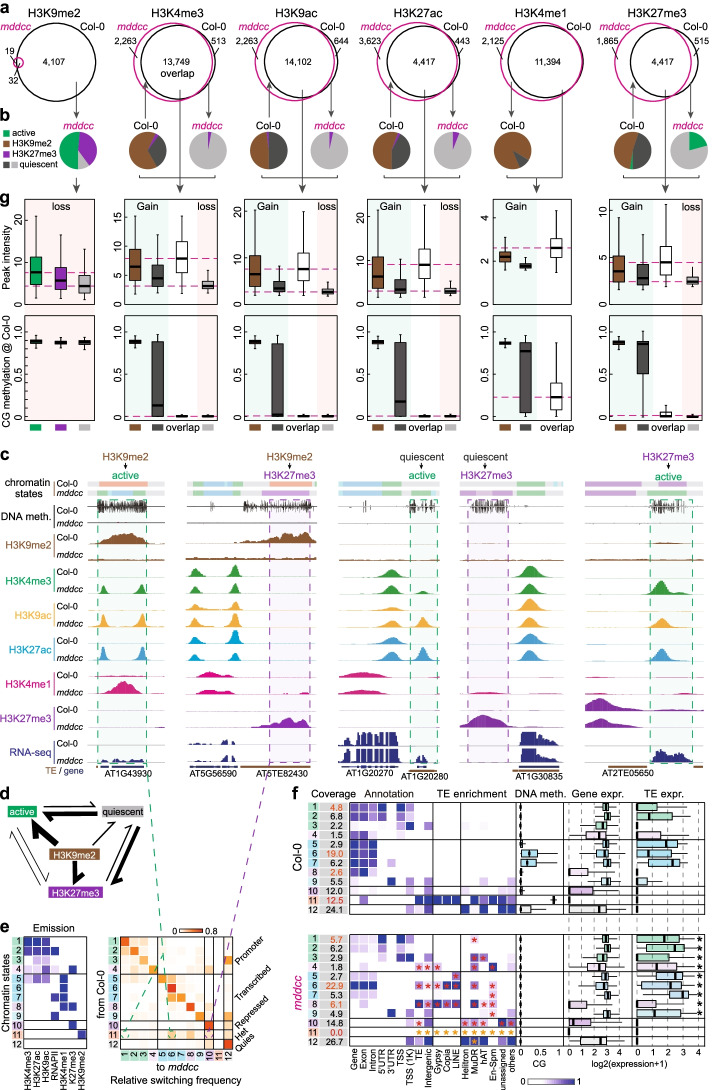
Complete loss of DNA methylation causes extensive conversions of histone modifications. **a** Overlap of histone modifications in *mddcc* and the wild type. **b** Conversion patterns of histone modifications between *mddcc* and the wild type. **c** Examples of histone modification conversions. **d** Histone modification conversion model. The line thickness is proportional to the switching frequency. **e** Composition (emission probability) of six histone modifications and RNA polymerase II (RNAPII) occupancy, and ratio of the observed probability that a region switches from one chromatin state (row, the wild type) to another (column, *mddcc*). **f** Properties of chromatin states defined in *mddcc* and the wild type. Red and yellow asterisks indicate higher and lower enrichments in *mddcc* than in the wild type, respectively. Black asterisks indicate a significant difference in the expression of TEs in the same chromatin state between *mddcc* and the wild type. *p < 0.001 from Wilcoxon test. **g** Peak intensity and DNA methylation of the indicated categories in (**a** and **b**)

To investigate histone modification switches between *mddcc* and the wild type, we defined four categories of chromatin states: active (marked with H3K4me3, H3K9ac, H3K27ac, or H3K4me1), H3K9me2, H3K27me3, and quiescent (without the detected histone marks). Although the chromatin can switch between different states, we found that most conversions were from H3K9me2 and quiescent to active or H3K27me3 (Fig. 4a–d), consistent with the complete loss of H3K9me2 in *mddcc*. Further analysis revealed that the heterochromatin state (CS11) exhibited the highest variability within TEs, changing from heterochromatin to active or H3K27me3 state, while the classic bivalent (CS4) and quiescent (CS12) states switched to a H3K27me3 state (CS10) (Fig. 4c, e, f). Thus, histone modification conversions are extensive and directional at specific genomic regions following loss of DNA methylation.

The stable active marks or the repressive mark H3K27me3 in *mddcc* and the wild type exhibited the highest peak intensities, whereas the variable marks, especially those lost in the mutant, displayed low peak intensities (Fig. 4g). Newly gained active marks as well as H3K27me3 in *mddcc* mainly switched from the H3K9me2 or quiescent regions that exhibited high DNA methylation in the wild type (Fig. 4g). Thus, heavy DNA methylation, regardless of its association with H3K9me2, plays an important role in regulating the deposition of active marks and the repressive mark H3K27me3, consistent with the concept that the binding affinities of histone modification-related enzymes and transcription factors can be regulated by changes in the DNA methylation state at specific loci [40–42].

### Contribution of CG and non-CG methylation to chromatin state switches

**Fig. 5:**
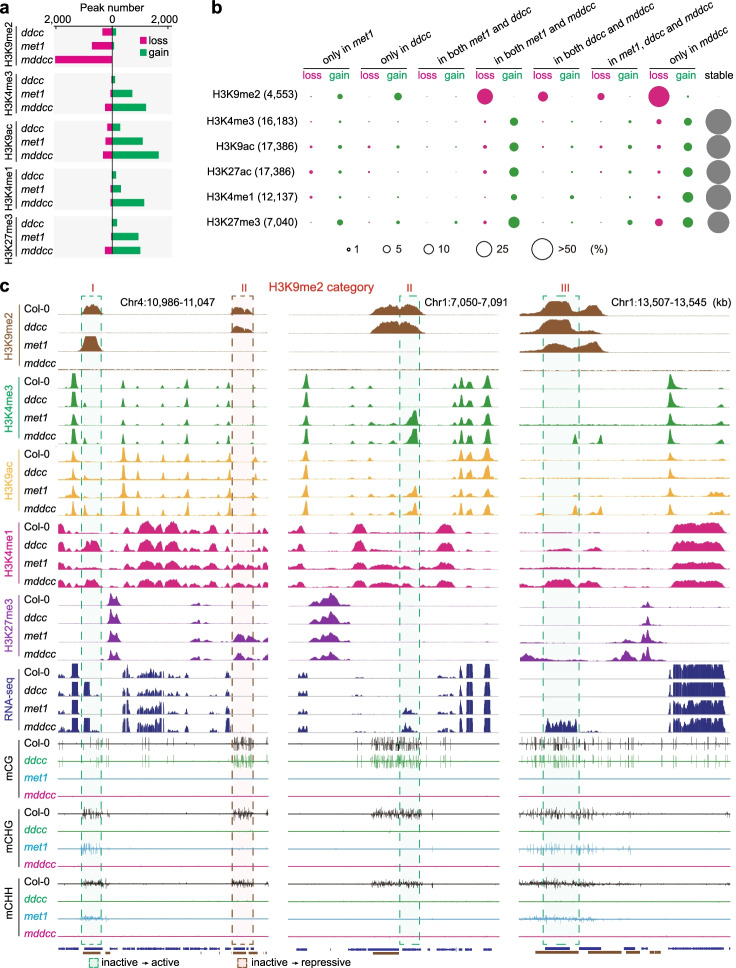
Dissecting the contribution of CG and non-CG methylation to the epigenomic landscape. **a** Numbers of changes of histone modification loci in *ddcc*, *met1*, and *mddcc* compared with the wild type. **b** Percentages of histone modification loci impacted by the indicated genotypes. The total number for a histone modification is the sum of the histone modification sites in the wild type and mutants. “Stable” indicates that the histone modifications retained in the mutants were similar to those in the wild type. The percentages of changes in a histone modification are represented by the area of the circle. **c** Representative examples of histone modification switches in the indicated genotypes. H3K9me2 categories were defined in Fig. 2a

To elucidate the relative contribution of CG and non-CG methylation to the histone modification landscape, we performed a comprehensive epigenomic analysis in *ddcc*, *met1*, and *mddcc*. Among the three mutants, *ddcc* had the least changes in active marks and the repressive mark H3K27me3, while *met1* had less changes compared with *mddcc*, consistent with the partial loss of H3K9me2 in *ddcc* and *met1* and total loss of H3K9me2 in *mddcc* (Fig. 5a). Compared with the total loss of H3K9me2 in *mddcc*, more than half of the active marks (~75%-82%) and H3K27me3 (~59%) was retained in all three mutants (Fig. 5b). This suggests that DNA methylation has a strong but relatively smaller effect on the distribution of active marks and H3K27me3 than H3K9me2 at the genome-wide level. There were thousands of ectopic active marks and H3K27me3 in the DNA methylation mutants (Fig. 5a). These ectopic marks were mainly enriched in two categories, “gain in both *met1* and *mddcc*” and “gain in *mddcc* only” (Fig. 5b, c), and thus were mainly affected by CG methylation and total C methylation. Collectively, our results suggest that CG and non-CG methylation independently regulate the chromatin state switches in some genomic regions but cooperatively regulate such switches in some other genomic regions.

### Role of histone modifications in regulating gene expression in the absence of DNA methylation

**Fig. 6:**
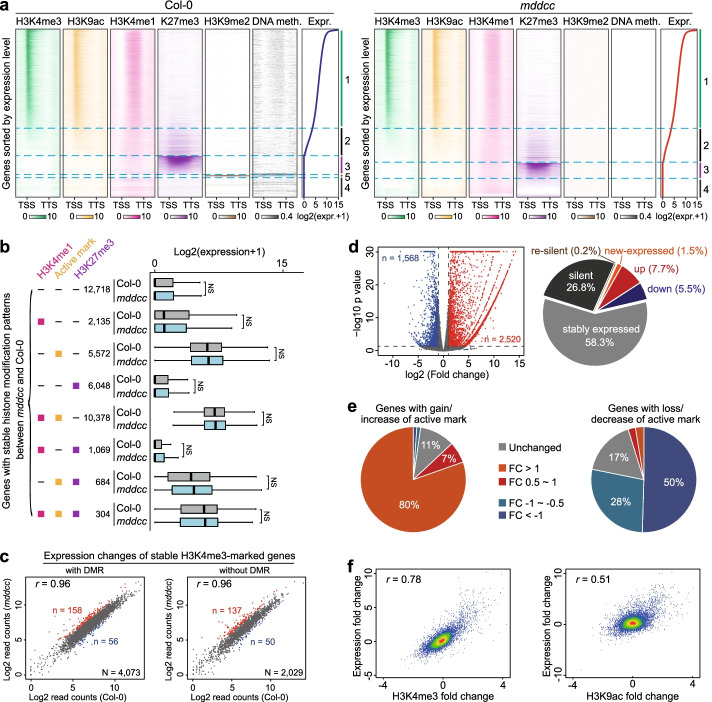
Role of histone modifications in the regulation of the expression of protein-coding genes in the absence of DNA methylation. **a** Distribution of epigenetic mark signals across the body (± 1 kb) of all protein-coding genes in the wild type and *mddcc*. Genes were sorted based on levels of expression, and genes with no detectable expression were sorted by H3K27me3 and H3K9me2 levels in the wild type (left panel) and *mddcc* (right panel), respectively. **b** Expression levels of genes with stable/unchanged histone modification patterns between the wild type and *mddcc*. Gene expression was standardized with TMM. **c** Dot plot depicting transcriptional changes in genes marked by stable H3K4me3 associated with or not associated with differentially methylated regions (DMR) in *mddcc* and the wild type. **d** Numbers and percentages of differentially expressed genes in *mddcc* compared with the wild type. **e** Expression fold change of genes with gain/increase and loss/decrease of active marks in *mddcc*. **f** Correlations of log2-fold change in gene expression and histone marks between *mddcc* and the wild type

Next, we explored the relationship between histone modifications and gene expression in the DNA methylation-free mutants. Except for a few genes marked by H3K9me2 in the wild type but not in *mddcc* (cluster 5), the majority of the genes were similarly marked in both *mddcc* and the wild type with active marks and/or H3K27me3 (clusters 1–3) (Fig. 6a). The relationship between histone modification patterns and gene expression levels for each cluster in *mddcc* is similar to that in the wild type (Fig. 6a). For example, in the DNA methylation-free mutant, genes containing active histone marks still tended to be highly expressed (cluster 1), bivalent marks correlated with moderately expressed genes (cluster 2), and H3K27me3 remained a repressive mark for silent genes (cluster 3) (Fig. 6a). No significant difference was detected in the expression of genes with stable histone modification patterns between *mddcc* and the wild type (Fig. 6b). Furthermore, most loci in which histone modifications remained stable in *mddcc* displayed similar expression levels in the wild type and *mddcc*, regardless of DNA methylation (Fig. 6c; Additional file 1: Fig. S12, S13). These results suggest that histone modifications exert similar effects on the regulation of gene expression in the wild type and *mddcc*. Collectively, our data indicate that histone modifications retained in DNA methylation-free plants, mainly active marks and H3K27me3, serve as DNA methylation-independent regulatory systems for gene transcription.

The number of differentially expressed genes was much larger in *mddcc* (4088; ~15%) compared with *ddcc* (545; ~2%) and *met1* (883; ~3%) (Fig. 4d; Additional file 1: Fig. S14a), suggesting that while CG methylation and non-CG methylation can regulate genome transcription independently, they cooperatively regulate many more genes. Most of these expression changes correlated with the corresponding changes in histone modifications: 71% of the upregulated genes were associated with gain/increase of active marks or loss/decrease of H3K27me3, while 64% of the downregulated genes were associated with loss/decrease of active marks or gain/increase of H3K27me3 (Additional file 1: Fig. S14b). When we examined this from a chromatin centric view, we found that the expression level of most genes with gain/increase of active marks were upregulated, whereas these with loss/decrease of active marks were down-regulated (Fig. 6e). These results, together with the high correlation between histone modification and gene expression changes (Fig. 6f), suggest that the transcriptional reprogramming observed upon loss of DNA methylation correlates with the redistribution of the examined histone modifications or through the modulation of the levels of histone modifications.

### Role of histone modifications in the control of TE activity upon loss of DNA methylation

**Fig. 7:**
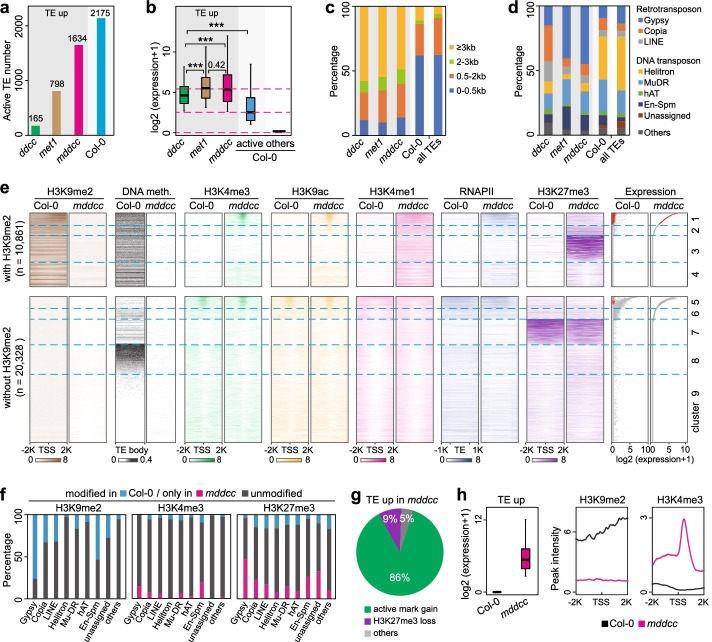
Roles of DNA methylation and histone modifications in the control of TE expression. **a** Number and percentage of derepressed TEs in the indicated genotypes. Number of active TEs in the wild type is shown. **b–d** Expression level (**b**), length distribution (**c**), and types (**d**) of derepressed TEs in the indicated genotypes. ***p < 2.6e−07 from Wilcoxon test. **e** Comparison of epigenetic patterns for TEs between *mddcc* and the wild type. Red dots, derepressed TEs in *mddcc*. **f** Percentage of TEs marked by different histone modifications in the wild type or only in *mddcc*. **g** Histone modification compositions of derepressed TEs in *mddcc*. **h** Comparison of transcript levels and epigenetic profiles for derepressed TEs in the wild type and *mddcc*. Gene expression was standardized with TMM

DNA methylation is involved in the control of TE activity; consistent with this, mutants lacking DNA methylation displayed a reactivation of TEs [8]. A higher number of TEs were derepressed in *met1* than in *ddcc*, suggesting that CG methylation plays a larger role than non-CG methylation in TE silencing [28] (Fig. 7a). Because of the cooperative effect of CG and non-CG methylation, there was a large increase in the number of derepressed TEs in *mddcc* [28] (Fig. 7a). Notably, in the wild type, more than 2000 TEs were active (Fig. 7a) and exhibited moderate expression levels (Fig. 7b); these TEs showed no obvious enrichment in length and types (Fig. 7c, d). These moderately expressed TEs were generally enriched in regions with low levels of DNA methylation and active marks but without H3K9me2 (Fig. 7e, cluster 5, grey dots), and probably had no harmful effect on normal plant development. In contrast, long TEs tended to be reactivated following depletion of DNA methylation, with the highest expression levels in *met1* and *mddcc*, followed by *ddcc* (Fig. 7b, c). Although both DNA transposons and retrotransposons were regulated by DNA methylation, there was an over-representation of Copia and LINE retrotransposons among those reactivated in *ddcc* [8] and of Gypsy and En-Spm among those reactivated in *met1* and *mddcc* (Fig. 7d). Hence, CG and non-CG methylation plays important roles in silencing different TE families (Additional file 3: Table S2).

We analyzed the chromatin features of TEs in *mddcc* and the wild type. All TEs were classified into 9 clusters, based on the epigenetic marks deposited and their transcript levels (Fig. 7e). Only one third of TEs were marked with H3K9me2 in the wild type (Fig. 7e, clusters 1–4), including most Gypsy and En-Spm and approximately one third of Copia and LINE (Fig. 7f). The majority of derepressed TEs in *mddcc* switched from heavy DNA methylation/H3K9me2 in the wild type to active marks in the mutant (Fig. 7e, cluster 1, red dots; Fig. 7g, h). For example, we identified 10 TE transposition events involving 4 TEs in *mddcc* in our previous study [28]*.* We examined the chromatin states of these 4 TEs and found that all of these TEs previously repressed with heavy DNA methylation and H3K9me2 in the wild type were marked by active marks and showed high levels of expression upon loss of total DNA methylation (Additional file 1: Fig. S15). In clusters 7–9, TEs showed epigenetic patterns similar to those in the wild type and remained silent except for the loss of DNA methylation in *mddcc* (Fig. 7e). Similarly, in cluster 4, TEs remained silent even though DNA methylation and H3K9me2 were lost in *mddcc* (Fig. 7e). These results indicate that removal of DNA methylation and H3K9me2 provides a permissive state for TE derepression, while ectopic gain of active marks is highly correlates with TE activation upon loss of DNA methylation.

Heavy DNA methylation/H3K9me2 and H3K27me3 are two independent, mutually exclusive epigenetic silencing mechanisms in both mammals and plants [17, 43]. In mammalian cells, the redistribution of H3K27me3 upon DNA hypomethylation results in gene de-repression [44–46]. Here, we observed that ~43% of the H3K9me2-marked TEs in the wild type were now repressed by H3K27me3 and remained silent or exhibited extremely low expression in *mddcc* (Fig. 7e, clusters 2 and 3; Fig. 4c). The switch from H3K9me2 to H3K27me3 was observed in all TE families (Fig. 7f), indicating an important role of H3K27me3 in repressing a large spectrum of TEs in response to DNA methylation loss. Interestingly, previous studies found an association between H3K27me3 and transposons that is partly retained in the early land plant *Marchantia polymorpha*, but the H3K27me3 is replaced by H3K9 methylation in flowering plants [47], contributing to the idea that H3K27me3 may be a more ancient transposon silencing system [48]. Collectively, these results add support to the concept that H3K27me3 and H3K9me2/heavy DNA methylation serve as two conserved genome defense mechanisms and complement each other to keep TEs silent and safeguard genome integrity in mammals and plants.

## Discussion

**Fig. 8:**
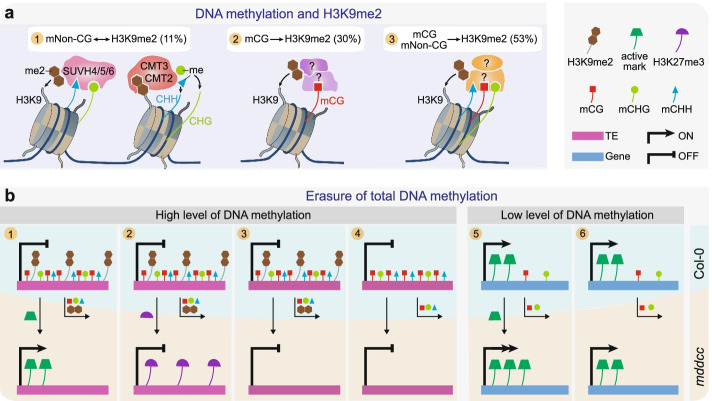
Function of DNA methylation in histone modification and transcription landscapes. **a** Distinct models of DNA methylation in regulating H3K9me2. (1) A feedback loop between non-CG methylation and H3K9me2: non-CG methylation recruits the H3K9 methyltransferases SUVH4, SUVH5 and SUVH6, and in turn, H3K9me2 facilitates CMT3 and CMT2 function to methylate CHG and CHH; (2 and 3) most of H3K9me2 is regulated by CG methylation (2) or by both CG and non-CG methylation (3), through yet unclear mechanisms. mCG, methylated CG; mNon-CG, methylated non-CG including methylated CHG and CHH. **b** Representative models for the impacts of DNA methylation on histone modifications and TE/gene transcription. Descriptions are in main text

DNA methylation and histone modifications coexist in the genome and interact with each other to form a complex code for chromatin regulation and transcription. This and previous studies [32] revealed that the connections between DNA methylation and H3K9 methylation are extensive but vary widely in different eukaryotes. Studies in the fungus *Neurospora crassa* [32, 49, 50] showed that this link is unidirectional, from H3K9 methylation to DNA methylation. In mammals, the relationship between these epigenetic marks is complex [32]: in some cases, DNA methylation is dependent on H3K9 methylation; in other cases, there is a self-reinforcing loop; yet in further cases, DNA and H3K9 methylation occur independently. In *Arabidops*is, previous research found a strong loss of H3K9me2 in *ddcc* and a weak loss in *met1*, leading to the widely known notion that there is a stronger dependency of H3K9me2 on non-CG methylation than on CG methylation [8]. Our results, however, differ from the previous work. One possibility is that the ChIP-seq method was unrefined at the time and the H3K9me2 ChIP-seq data [8] in the previous work was of very low quality and unreliable. In comparison, the eChIP-seq method [29] used here generated much higher quality ChIP-seq data and captured histone-modified loci with much greater sensitivity (Additional file 2: Table S1d). Our work supports the essential role of DNA methylation in the regulation of H3K9 methylation and revealed complex functional links between these two marks, as shown in Fig. 8a: in a small proportion of loci, H3K9me2 is subjected to a self-reinforcing loop with non-CG methylation [18, 19, 32]; more frequently, H3K9me2 is dependent on CG methylation; and more than half of H3K9 methylated loci are regulated cooperatively by CG and non-CG methylation. The exact regulatory mechanisms of the latter two links are to date unclear in plants. In future studies, it will be of great interest to determine the histone-modifying enzymes that recognize methylated DNA to ensure the establishment of H3K9me2 in specific sequence contexts.

The *mddcc* mutant completely devoid of DNA methylation enabled the investigation of the genome-wide effect of lack of DNA methylation on the histone modification landscape and transcription reprogramming. Our results demonstrate that transcriptional reprogramming resulting from the loss of DNA methylation is largely associated with the redistribution of the examined histone modifications and switches of chromatin states. Several representative regulatory models are summarized here (Fig. 8b). The majority of heavy DNA methylation regions coincide with TEs (Fig. 7e). In some cases, removal of high levels of DNA methylation in TEs causes a chromatin state switch from H3K9me2 to active marks or H3K27me3, correlating with the activation or continued repression of TEs, respectively (Fig. 8b, cases 1 and 2). All TE transposition events identified in *mddcc* [28] belong to the first category (Fig. 8b, case 1), suggesting that chromatin state transition switches, especially between inactive and active states, may participate in TE transposition and genome integrity in mammals and plants. In other cases, TEs with heavy DNA methylation, regardless of H3K9me2 deposition, remain silent in the DNA methylation mutants even though there is no H3K27me3 mark (Fig. 8b, cases 3 and 4), indicating that some undetected repressive marks and/or uncovered regulatory mechanisms may exist in these regions. Compared with TEs, most genes display no or low levels of DNA methylation in the wild type (Fig. 6a; Additional file 1: Fig. S1). After removal of DNA methylation, the expression of most genes is not affected, and the differential expression of a small proportion of genes may result from switches or increase/decrease of histone modifications (Fig. 6e and Fig. 8b, case 5; Additional file 1: Fig. S14). Collectively, our data indicate that the function of DNA methylation in the regulation of gene/TE expression varies in a locus-dependent manner based on the abundance of this mark and the specific target sequences.

Our results suggest that histone modifications, especially active marks and H3K27me3, play a central role in the control of genome activity of plants in the absence of DNA methylation. Histone modifications also play a central role in genome activity in the DNA methylation-free organism *Caenorhabditis elegans* [51–53]. One important difference, however, is that both constitutive and facultative heterochromatin, marked with H3K9me2/3 and H3K27me3, respectively, coexist in *C. elegans*, whereas only H3K27me3, which perhaps partly replaces the functions of H3K9me2, is preserved in plants without DNA methylation. Ectopic gain of H3K27me3 in *mddcc* at some loci previously marked by DNA methylation and/or H3K9me2 (Fig. 4c, Fig. 7e) implies that DNA and H3K9 methyltransferases have stronger affinities than the H3K27me3 methyltransferases at these loci in the wild type. The chromatin environment lacking DNA methylation and H3K9me2 in *mddcc* mutant probably facilitates the recruitment of Polycomb Repressive Complex 2 to deposit the H3K27me3 mark at these loci. Hence, histone modifications not only can combine with DNA methylation to form a complex epigenetic regulatory code but also can serve as DNA methylation-independent regulators of genome activity.

## Conclusions

Taken together, this study not only provides an invaluable resource for the epigenetics research community but also yield new insights into the contribution of DNA methylation to the plasticity of the epigenetic landscape in *Arabidopsis*, particularly to the establishment and/or redistribution of histone modifications, and the roles and hierarchy of these different epigenetic marks in the control of genome transcription.

## Methods

### Plant materials

All mutant lines used in this study are of the Columbia ecotype. *met1* [9], *ddcc* and *mddcc* [28] mutants were previously described. *mddcc* quintuple mutant plants were identified and isolated from *met1/+ drm1 drm2 cmt2 cmt3*. Plants were grown at 22 °C under a 16 h light/8 h dark photo-period. The aerial part of 2-week-old plants for the wild type, *met1,* and *ddcc*, and the aerial part (with the inflorescence removed) of 5-week-old plants for the wild type, *met1, ddcc*, and *mddcc* were used for all experiments.

### ChIP-seq and data analysis

Tissues were cross-linked with 1% formaldehyde and quenched with 0.2 M glycine at room temperature. Approximately 0.01–0.1 g of tissue were used for each ChIP-seq assay as reported [29, 37] with minor modifications. Briefly, tissues were ground in liquid nitrogen into fine powder, lysed with Buffer S [50mM HEPES-KOH (pH7.5), 150mM NaCl, 1mM EDTA, 1% Triton X-100, 0.1% sodium deoxycholate, and 1% SDS] for 20 min at 4 °C and then mixed with Buffer F (Buffer S without SDS) for 10 min at 4 °C. The chromatin was fragmented to 200–600 bp through sonication. The lysates were centrifuged at 20,000 × *g* for 5 min at 4 °C, and the supernatant was collected for ChIP. The fragmented chromatin was incubated with antibodies against H3K4me1 (ABclonal, A2355), H3K4me3 (ABclonal, A2357), H3K9ac (Abcam, ab4441), H3K9me2 (Abcam, ab1220), H3K27ac (ABclonal, A7253), H3K27me3 (ABclonal, A2363), and RNAPII (BioLegend, 920102). The specificity of antibodies was verified prior to their use [29]. Antibodies were added to Dynabeads® protein G beads (Life Technologies, 10003D) and incubated for 6 h with rotation at 4 °C. The beads were washed with PBST twice and incubated with the fragmented chromatin overnight with rotation at 4 °C. The beads were washed subsequently with low-salt buffer (50 mM HEPES-KOH, 150 mM NaCl, 1 mM EDTA, 1% Triton X-100, 0.1% sodium deoxycholate, 0.1% SDS), high-salt buffer (low salt ChIP buffer replaced with150 mM NaCl with 350 mM NaCl), ChIP wash buffer (10 mM Tris-HCl pH 8.0, 250 mM LiCl, 0.5% NP-40, 1 mM EDTA, and 0.1% sodium deoxycholate), and TE buffer (10 mM Tris-HCl, pH 8.0, and 1 mM EDTA). The protein–DNA complexes were eluted from the beads by adding 100 μl of Elution buffer (50 mM Tris-HCl pH 7.5, 10 mM EDTA, and 1% SDS) for 15 min at 65 °C on a Thermomixer with rotation (900 rpm). Next, 5 μl of proteinase K were added to the eluted protein–DNA complexes, which were mixed and incubated at 55 °C overnight for reverse cross-linking. ChIP DNA was extracted and resuspended in TE buffer. Libraries were prepared using an NEBNext® Ultra™ II DNA library prep kit for Illumina® sequencing (New England BioLabs, E7645) and sequenced using an Illumina HiSeq X Ten platform (paired-end 150-bp reads).

Raw reads were first trimmed as paired-end reads using Fastp [54] with default parameters. Clean reads were mapped to the TAIR10 *Arabidopsis* genome by BWA-MEM with default parameters. SAMtools [55] was used to remove redundant reads and align low quality (map quality < 30) reads. Next, ChIP-seq peaks were called by MACS2 [56], broad peak with parameters “-f BAMPE -B --broad -g 119667750 -q 0.00001 --broad-cutoff 0.00001” and narrow peak with parameters “-f BAMPE -B -g 119667750 -q 0.00001.” The mapped BAM files were normalized and converted to BigWig format using Deeptools [57] to configure the tracks in IGV [58]. Next, we defined the peaks that met the following criteria as the newly detected peaks: (1) no peak was detected in the wild type using MACS2, and a peak was detected in the mutant; (2) the peak intensity in the mutant was two-fold higher than that in the wild type; and (3) in the mutant, the peak signal was greater than 1 after subtracting the corresponding input signal. We used the same computational criteria to define the missing peaks in the mutant. Differential peak analysis was performed with Deeptools (computeMatrix) with a threshold fold change of >1.5.

### Heatmaps and profiles

The heatmap and peak profile were generated by Deeptools. First, ComputeMatrix was used to calculate the peak density distribution matrix, and then the corresponding visualization results were generated by PlotHeatmap and PlotProfile. The heatmap and profile related to methylation were generated using BatMeth2. The methylation level file was generated by using methyGff, and then bt2profile.py and bt2heatmap.py scripts were used for visualization. The results shown in Fig. 3c are the heatmap of H3K4me1 peak center and the upstream and downstream 2Kb. Considering that the distribution position of active marks (H3K4me3, H3K9ac, H3K27ac) is not directly coincident with H3K4me1, but localizes around it, we defined the active mark within 500 bp upstream and downstream of H3K4me1 as H3K4me1-related active marks.

### Whole-genome bisulfite DNA sequencing (BS-seq) and data analysis

Genomic DNA was extracted using the DNeasy Plant Mini Kit (QIAGEN, 69104). Bisulfite conversion of DNA was conducted using the EZ DNA Methylation-Gold™ kit (ZYMO, D5005), and the bisulfite-treated DNA libraries were constructed using the Illumina TruSeq DNA sample prep kit, following the manufacturer’s instructions. The libraries were then sequenced using an Illumina HiSeq X Ten platform (paired-end 150-bp reads). For data analysis, low-quality read trimming and artificial sequence trimming were performed using Fastp with default parameters. Clean reads were mapped to the *Arabidopsis* genome (TAIR10) using BatMeth2-align [59] with default parameters. DNA methylation calling was performed with BatMeth2-calmeth with parameter ‘-n 0.1 -Q 30’, and the SAM file was converted to the BAM format with SAMtools [55]. We then used batmeth2_to_bigwig.py scripts to generate BigWig files for visualization using IGV.

### Total RNA-seq and data analysis

Total RNA was isolated using the RNeasy Plant Mini Kit (QIAGEN, 74904); library preparation was performed at the PSC Genomics Core Facility according to the manufacturer’s instructions (Illumina). The libraries were sequenced on an Illumina HiSeq X Ten platform (paired-end 150-bp reads). Raw reads were first trimmed as paired-end reads using Fastp with default parameters to remove the adaptors and low-quality reads. Clean reads were mapped to the *Arabidopsis* genome (TAIR10) using Hisat2 [60] with default parameters, and then SAMtools was used to sort the BAM file and remove the low map quality reads with -q 30. Reads counts per gene were generated using HTSeq-count [61]. The differential expression test was performed using DESeq2 [62] with the threshold |log2 fold change| > 1 and q value < 0.01. In order to perform gene expression comparisons between and within samples, we used EdgeR’s trimmed mean of M-values (TMM) method for normalization [63, 64]. The mapped BAM files were normalized and converted to BigWig format using Deeptools to configure the tracks in IGV.

### Redefinition of H3K9me2 loci

We observed that the peak length of a certain proportion of H3K9me2 is greater than 5 kb or even 10 kb (Additional file 1: Fig. S4a), whereas the length of most H3K4me3, H3K9ac and gene is only 1–2 kb. Therefore, for better follow-up analysis, we split H3K9me2 peak according to the distribution of genes, TE, and other modifications on H3K9me2 peak. The specific criteria are as follows. As shown in Additional file 1: Fig. S4b, H3K9me2 with a peak length less than 5 kb was retained as a H3K9me2 locus without segmentation. Peaks longer than 5 kb were required to be further segmented. Briefly, histone-modified regions (except H3K9me2) and gene (protein coding gene and TE gene) regions were first merged using BEDTools [65]. Next, H3K9me2 with a peak longer than 5 kb was segmented to generate new loci with a length less than 5 kb by overlapping with data in the aforementioned merged documents. Then, the remaining H3K9me2 with a peak longer than 5 kb was divided into new loci with an equal length (≤ 5 kb). Finally, the aforementioned loci were merged into a new H3K9me2 loci file.

## Supplementary Information

**Additional file 1: Figure S1.** Dissecting contributions of DNA methyltransferases to DNA methylation patterns in five-week-old plants. Figure S2. Similar histone modification patterns between two- and five-week-old plants for the indicated genotypes. Figure S3. Global view of gain/loss of histone modifications and differentially expressed genes. Figure S4. Redefinition of H3K9me2 loci. Figure S5. The “Overlapping” H3K9me2 signals in *mddcc* are likely noises. Figure S6. The “*mddcc* specific” H3K9me2 signals are likely noises. Figure S7. Similar patterns of histone modifications and expression of *SUVH* and *SUVR* genes between Col-0 and *mddcc*. Figure S8. Impacts of CG and non-CG methylation on the deposition of H3K9me2. Figure S9. DNA methylation is required for proper *IBM1* expression. Figure S10. Heatmap representation of histone marks and DNA methylation changes in the wild type and indicated mutants. Figure S11. Chromatin states, genomic annotation, TE enrichment, DNA methylation, and gene and TE expression in the indicated genotypes. Figure S12. Dot plot depicting the transcript changes in genes marked by stable histone marks associated with or not associated with differentially methylated regions (DMR) in *mddcc* and the wild type. Figure S13. Examples of no significant effect of DNA methylation on the expression of genes with stable histone modifications. Figure S14. Relationship between histone modifications and expression of protein-coding genes in DNA methylation-free mutants. Figure S15. The epigenetic patterns of transposed TEs in *mddcc*.

**Additional file 2: Table S1.** Summary of transcriptome and epigenome data in this study.

**Additional file 3: Table S2.** List of upregulated Tes with gain of active marks in *ddcc*, *met1*, and *mddcc* mutants.

**Additional file 4:** Review history.

## Data Availability

All sequence data have been deposited in NCBI GEO accession codes GSE183987 [66] and GSE169497 [28]. The script and result file were uploaded to Zenodo, and the data link is 10.5281/zenodo.6575422 [67]. All data supporting the findings of this study are available within the manuscript and its supporting information or are available from the corresponding author upon request.
