# Supplementary material for: DNA methylation underpins the epigenomic landscape regulating genome transcription in Arabidopsis
Source: Genome Biol. 2022 Sep 20;23:197. doi: 10.1186/s13059-022-02768-x (PMC9487137; doi:10.1186/s13059-022-02768-x)
Supplement: Supplementary file 1 — Additional file 1: Figure S1. Dissecting contributions of DNA methyltransferases to DNA methylation patterns in five-week-old plants. Figure S2. Similar histone modification patterns between two- and five-week-old plants for the indicated genotypes. Figure S3. Global view of gain/loss of histone modifications and differentially expressed genes. Figure S4. Redefinition of H3K9me2 loci. Figure S5. The “Overlapping” H3K9me2 signals in mddcc are likely noises. Figure S6. The “mddcc specific” H3K9me2 signals are likely noises. Figure S7. Similar patterns of histone modifications and expression of SUVH and SUVR genes between Col-0 and mddcc. Figure S8. Impacts of CG and non-CG methylation on the deposition of H3K9me2. Figure S9. DNA methylation is required for proper IBM1 expression. Figure S10. Heatmap representation of histone marks and DNA methylation changes in the wild type and indicated mutants. Figure S11. Chromatin states, genomic annotation, TE enrichment, DNA methylation, and gene and TE expression in the indicated genotypes. Figure S12. Dot plot depicting the transcript changes in genes marked by stable histone marks associated with or not associated with differentially methylated regions (DMR) in mddcc and the wild type. Figure S13. Examples of no significant effect of DNA methylation on the expression of genes with stable histone modifications. Figure S14. Relationship between histone modifications and expression of protein-coding genes in DNA methylation-free mutants. Figure S15. The epigenetic patterns of transposed TEs in mddcc. [file 13059_2022_2768_MOESM1_ESM.pdf]

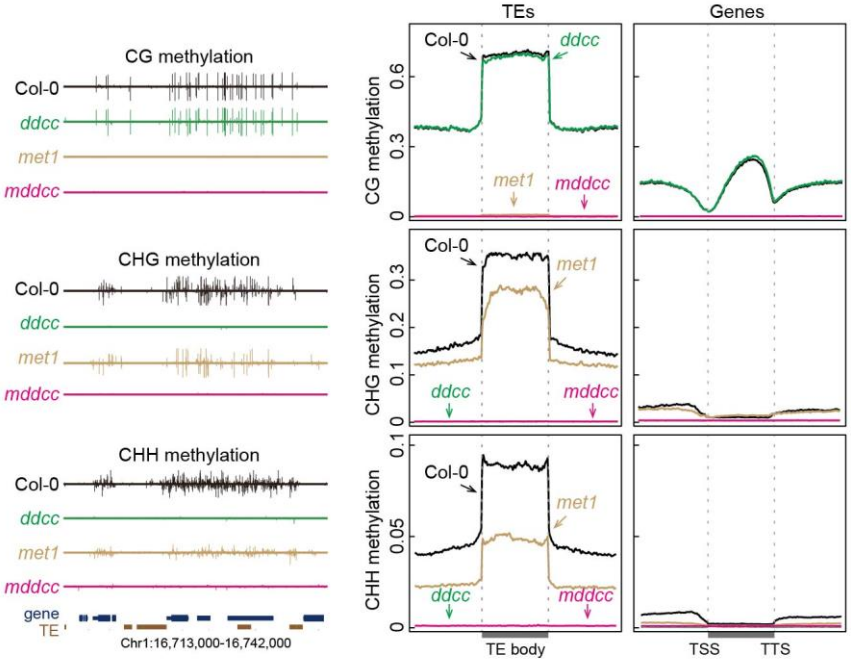

Fig. S1. Dissecting contributions of DNA methyltransferases to DNA methylation patterns in five-week-old plants.

Genome browser view and average distribution of CG, CHG, and CHH methylation over TEs and genes in five-week-old plants of the indicated genotypes. Flanking regions are 2 kb upstream and downstream of the gene or TE body. TSS, transcription start site. TTS, transcription termination site.

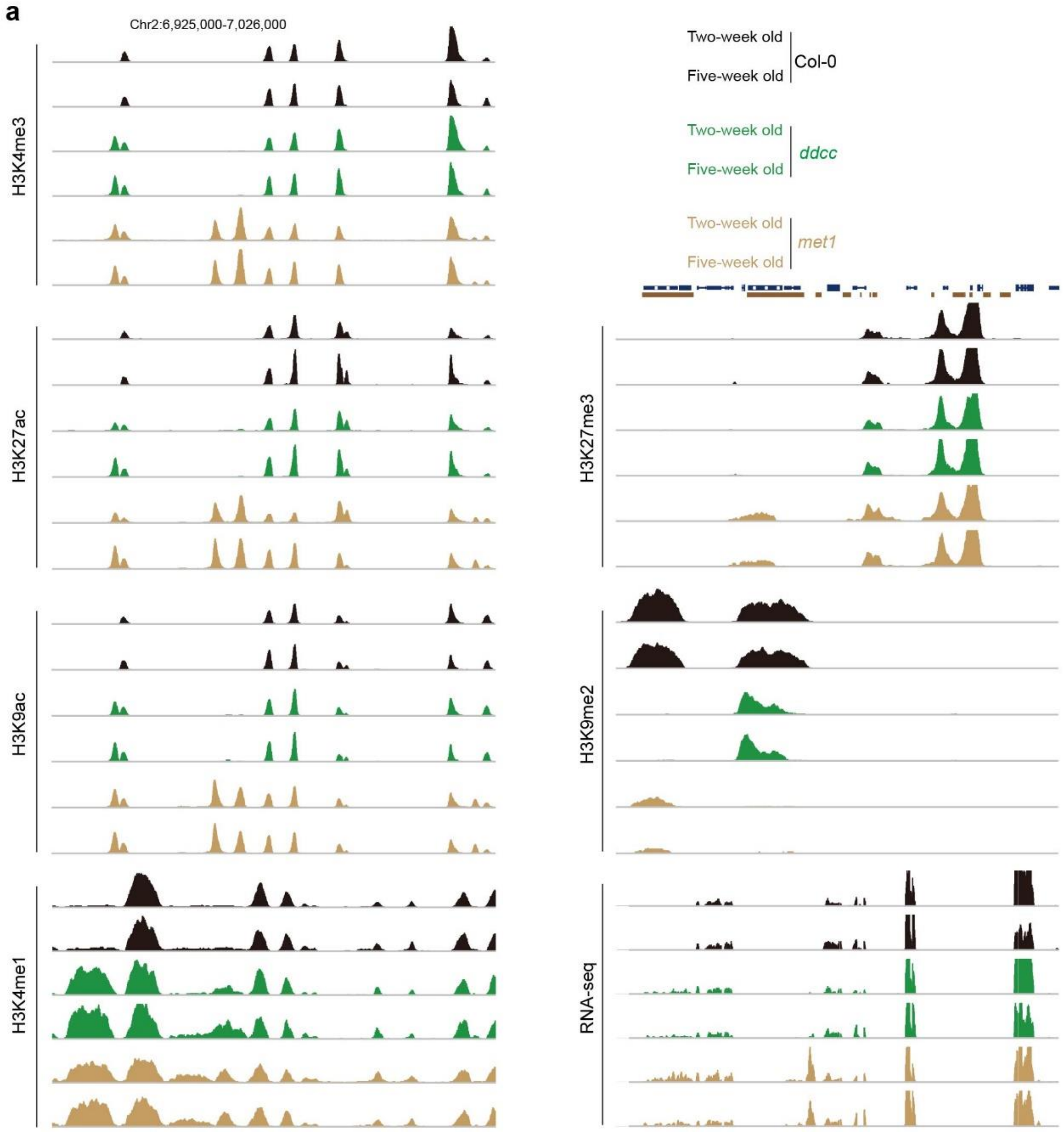
a
Chr2:6,925,000-7,026,000
Two-week old
Five-week old
Col-0
Two-week old
Five-week old
ddcc
Two-week old
Five-week old
met1
H3K4me3
H3K27ac
H3K9ac
H3K4me1
H3K27me3
H3K9me2
RNA-seq

Continued

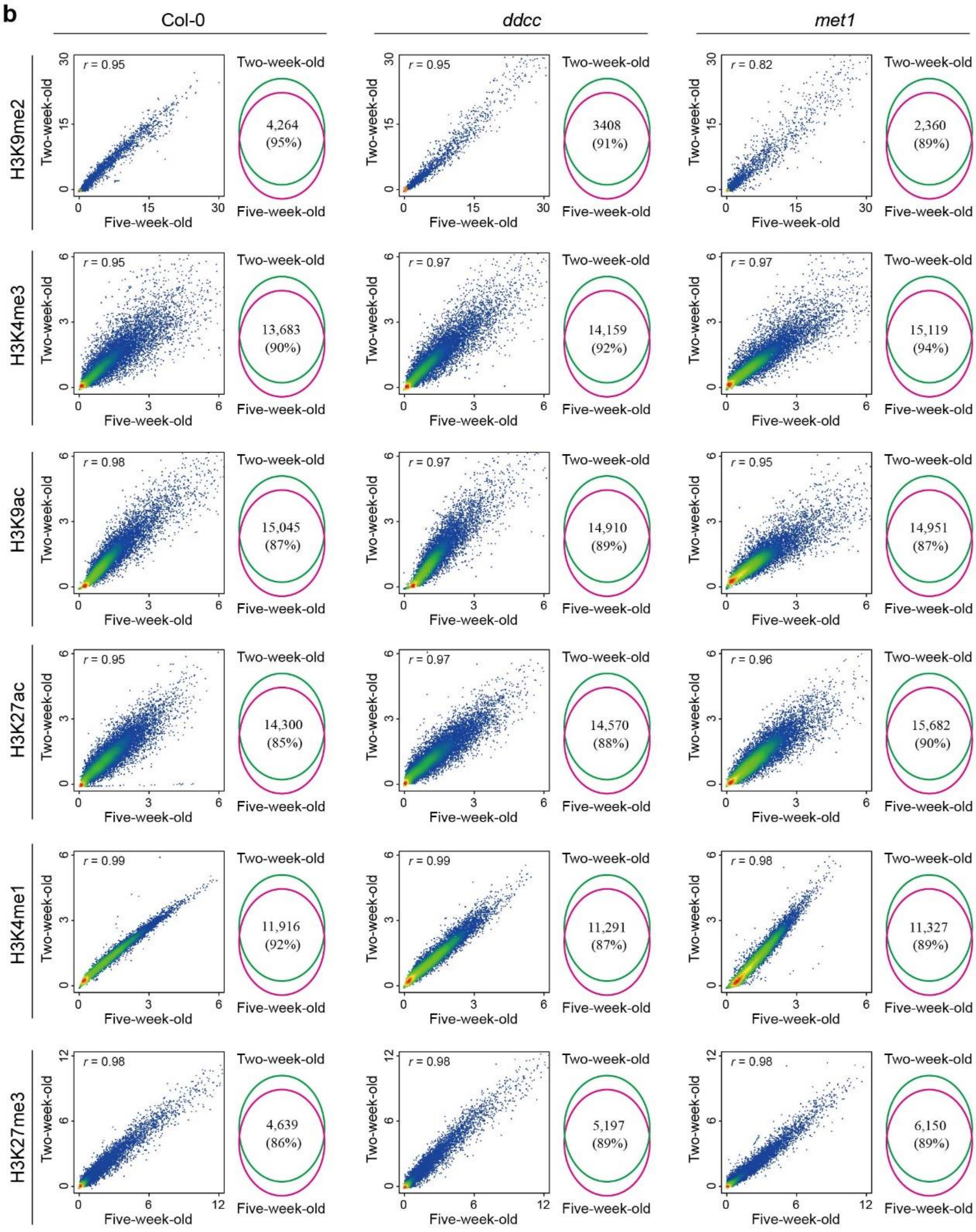

Fig. S2. Similar histone modification patterns between two- and five-week-old plants for the indicated genotypes.

a, Genome browsers of histone modifications and gene expression in two- and five-week-old plants. **b,** Correlations of histone modifications between two- and five-week-old plants. Dot plots depict log of mapped reads from each genomic bin (100 kb) between two- and five-week-old plants. Spearman’s correlation is shown. Venn diagrams display high percentages of overlapping peaks between two- and five-week-old plants.

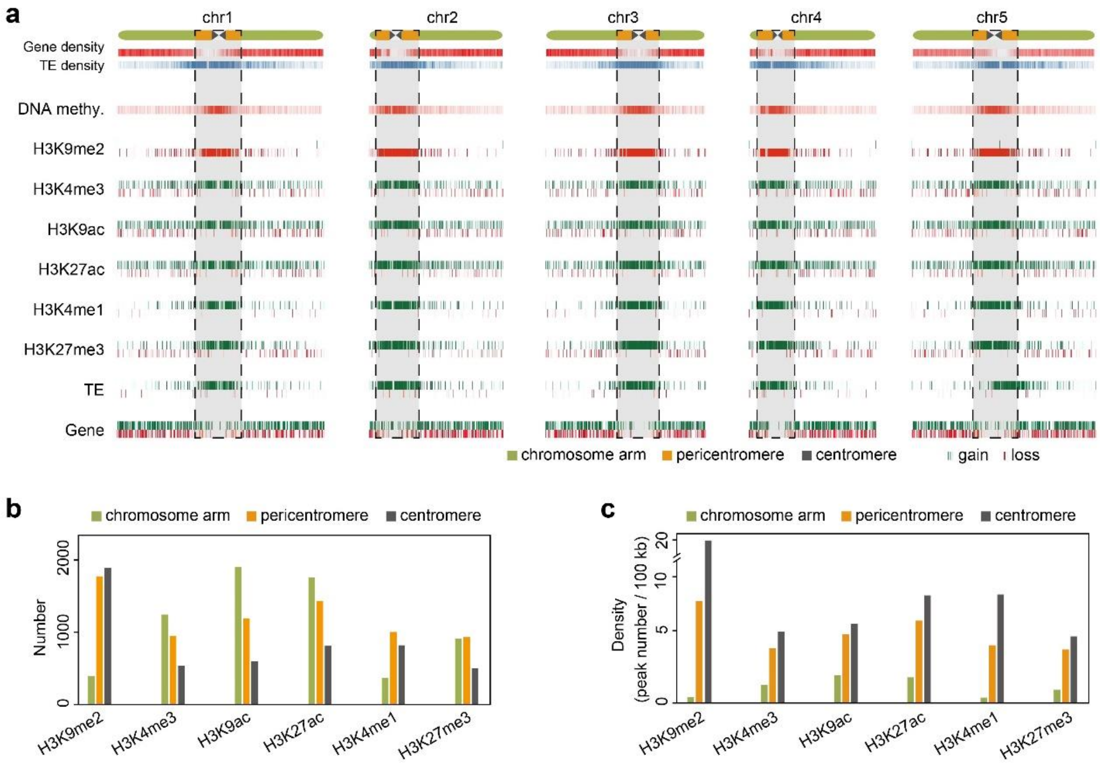

Fig. S3. Global view of gain/loss of histone modifications and differentially expressed genes. **a,** Chromosomal views of gain/loss of histone marks and genome transcription in *mddcc* compared with the wild type. Regions of chromosome arm, pericentromere, and centromere are indicated. **b**, **c,** Number (**b**) and density (**c**) of the gain/loss of histone modifications in the indicated regions.

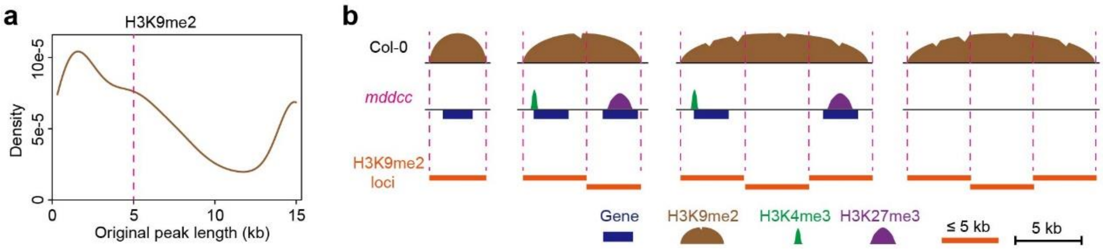

Fig. S4. Redefinition of H3K9me2 loci.

a, Density of original H3K9me2 peak length called by MACS2. **b,** Redefinition of H3K9me2 loci based on the indicated switching modes and original H3K9me2 peak length (see Methods for details).

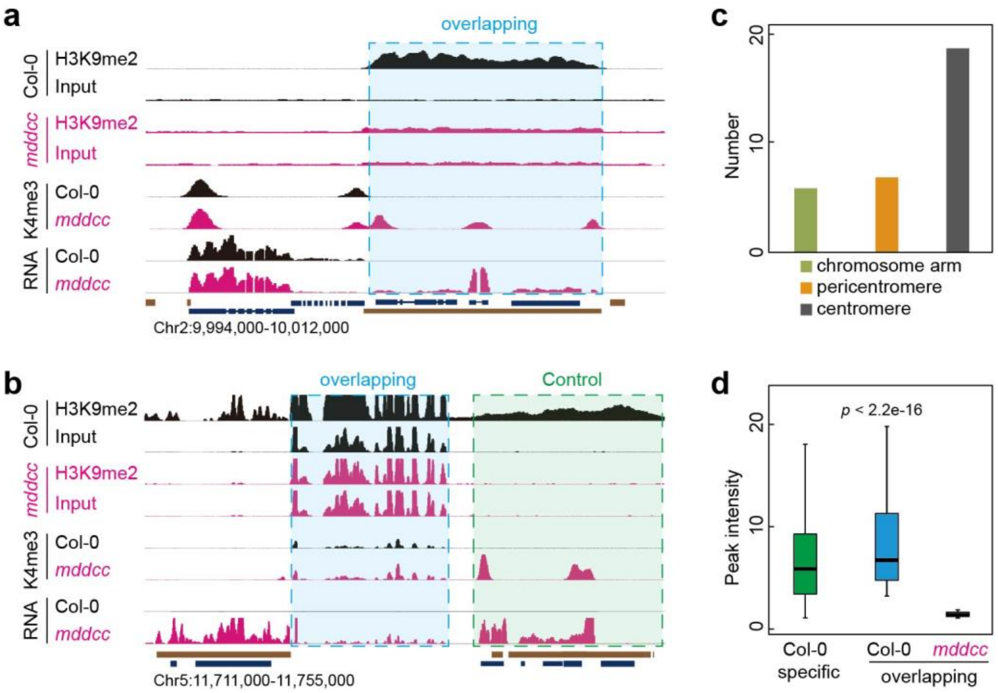

Fig. S5. The "Overlapping" H3K9me2 signals in *mddcc* are likely noises.

a and **b,** Representative genome browser views of "overlapping" H3K9me2 peaks between Col-0 and *mddcc* in Fig. 2a (Type VII). In contrast to canonical H3K9me2 (Control, green shadow), relatively low/similar signals were found in both H3K9me2 ChIP-seq and input at the "overlapping" H3K9me2 loci. Moreover, RNA-seq signals were only observed in the "overlapping" H3K9me2 loci of *mddcc*. **c,** Distribution of the "overlapping" H3K9me2 loci in the genome. **d,** Peak intensity of the "overlapping" H3K9me2 of *mdd*cc. The *p* value was calculated by Student's *t* test.

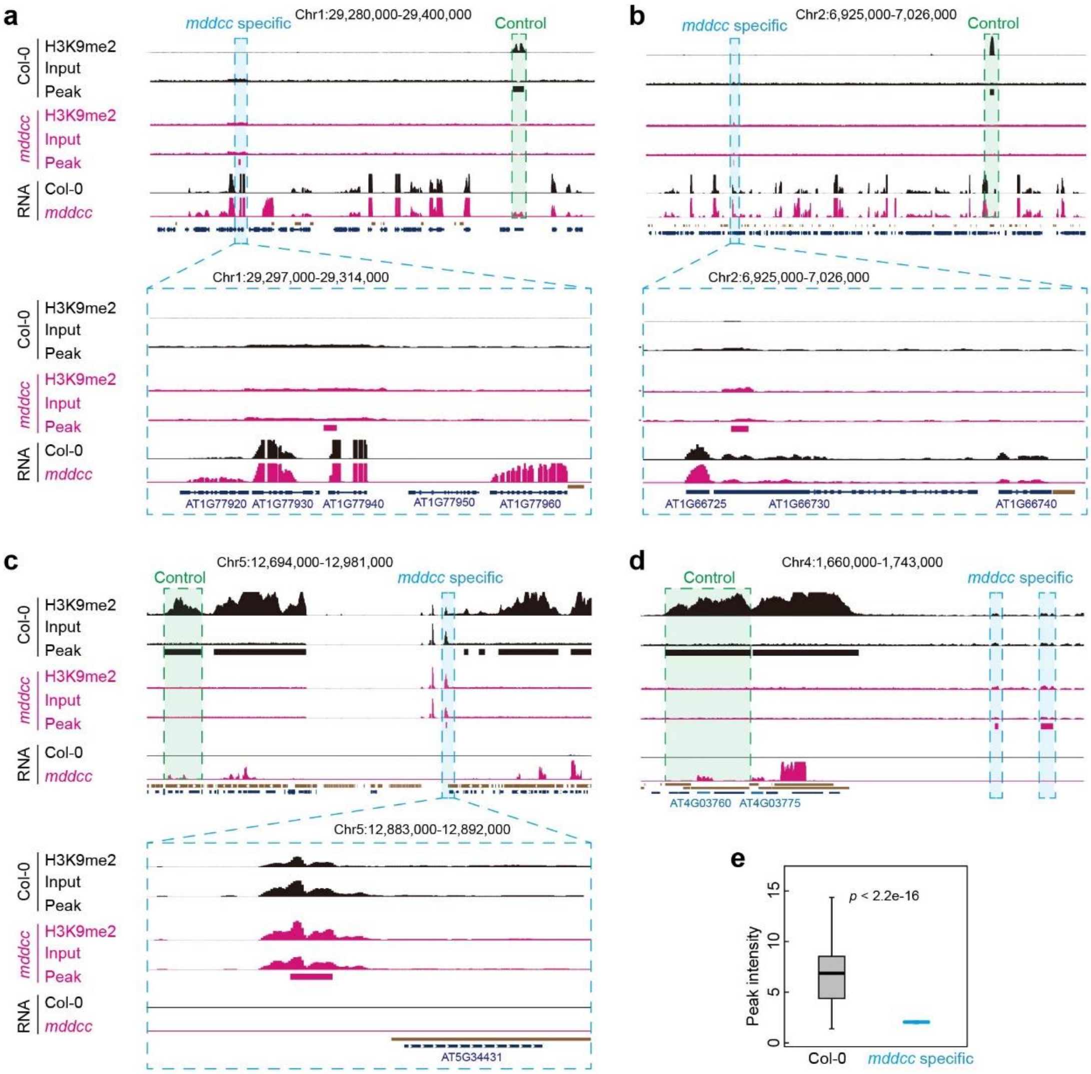

Fig. S6. The "*mddcc* specific" H3K9me2 signals are likely noises.

a–d, Representative screenshots of "*mddcc* specific" H3K9me2 peaks in Fig. 2a (Type VIII). BroadPeak file generated from MACS2 displays the called peaks (black and red bars). In contrast to the canonical H3K9me2 (Control, green shadow), low signals appeared in both H3K9me2 ChIP-seq and input at the "*mddcc* specific" H3K9me2 loci. Similar RNA-seq signals were observed at these loci of *mddcc* and Col-0. **e,** Peak intensity comparison for H3K9me2 in Col-0 and only in *mddcc*. The *p* value was calculated by Student's *t* test.

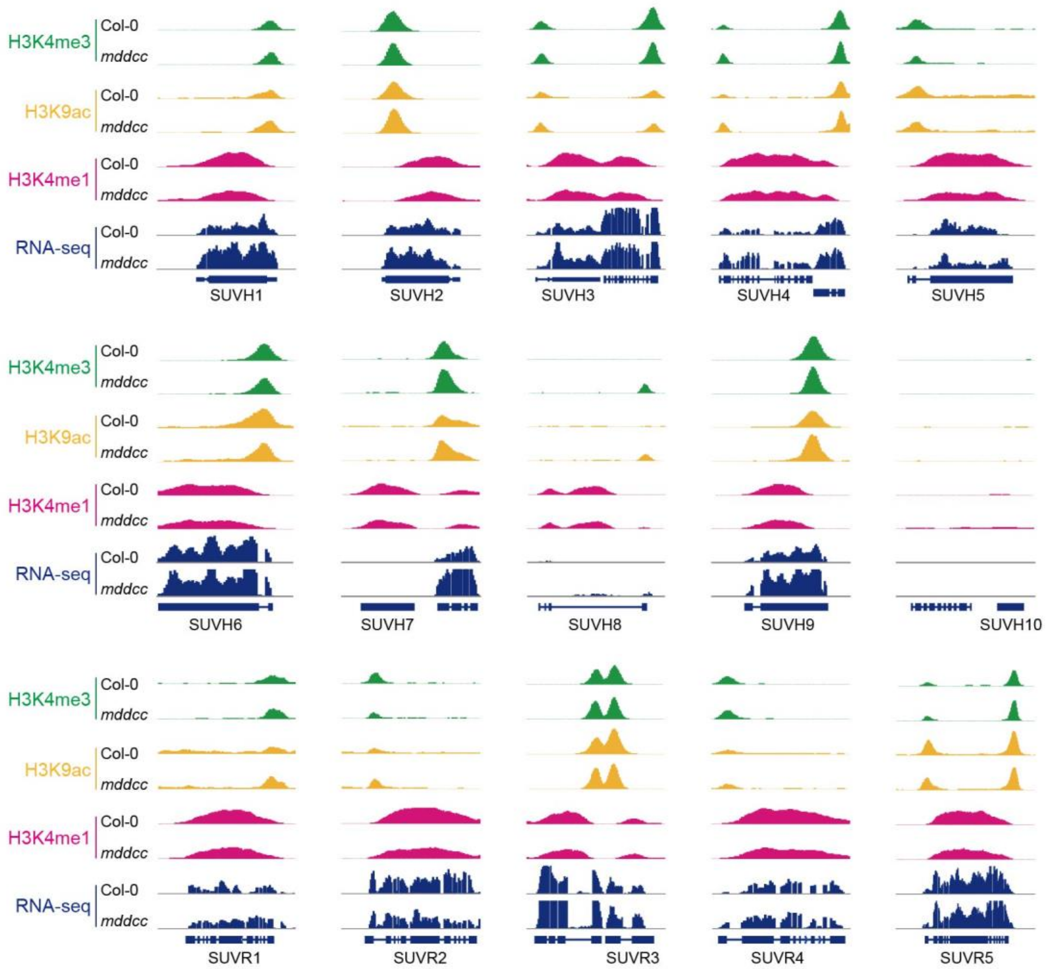

Fig. S7. Similar patterns of histone modifications and expression of *SUVH* and *SUVR* genes between Col-0 and *mddcc*.

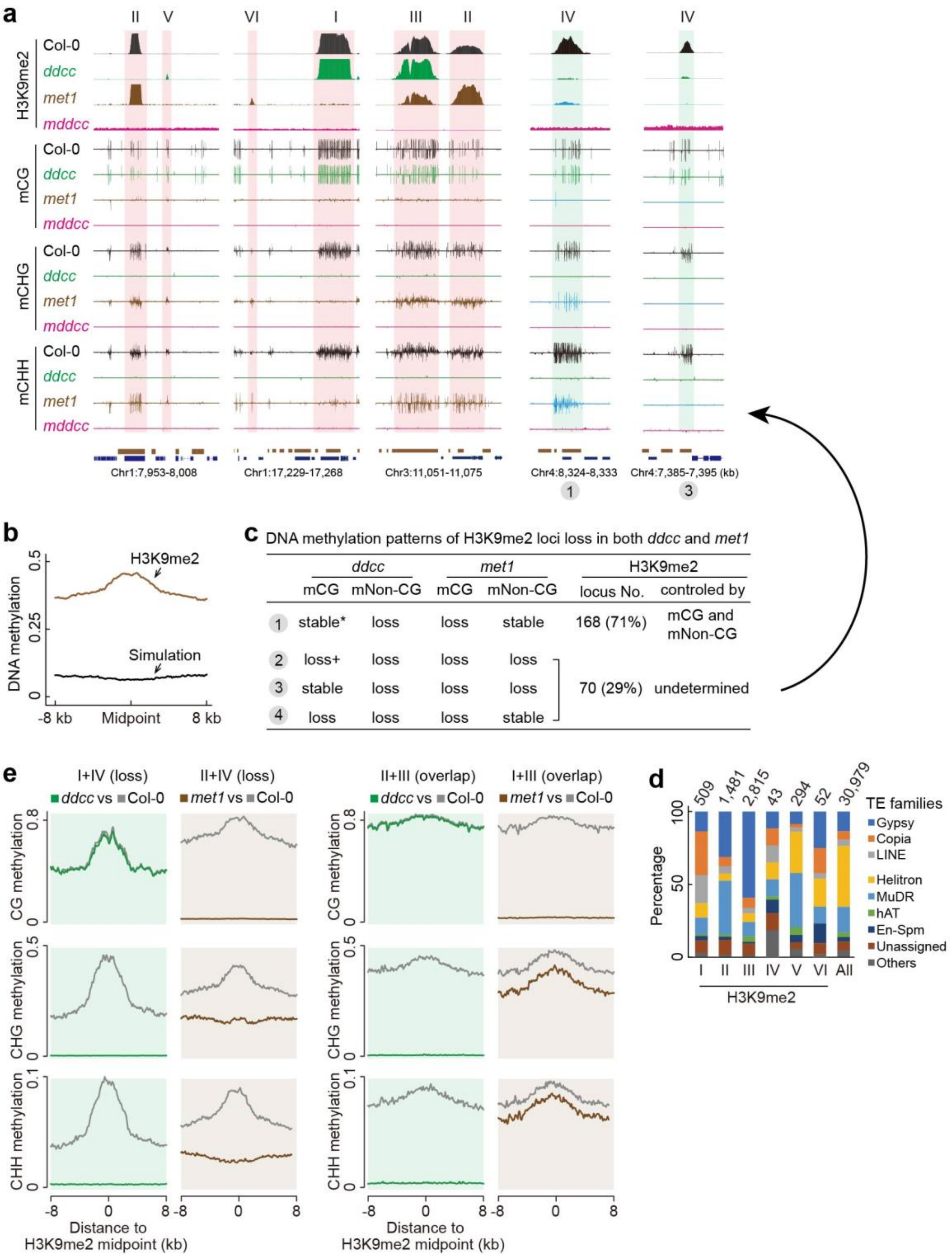

Fig. S8. Impacts of CG and non-CG methylation on the deposition of H3K9me2.

a, Representative examples of the indicated types of H3K9me2 marks. **b,** DNA methylation surrounding H3K9me2 in the wild type. Simulation indicates randomly selected genomic regions without H3K9me2. **c,** DNA methylation patterns of H3K9me2 loci lost in both *ddcc* and *met1*. Most (71%) of these H3K9me2 loci are controlled by CG and non-CG methylation separately. *Stable means that there is no significant change in DNA methylation in the specific region. +Loss

means more than 50% reduction of DNA methylation in specific region in mutant compared with the wild type. mCG, methylated CG; mNon-CG, methylated non-CG including methylated CHG and CHH. **d,** Types of TEs marked by the indicated types of H3K9me2. **e,** Distribution of DNA methylation at the indicated categories of H3K9me2 loci in *ddcc* and *met1* compared with the wild type.

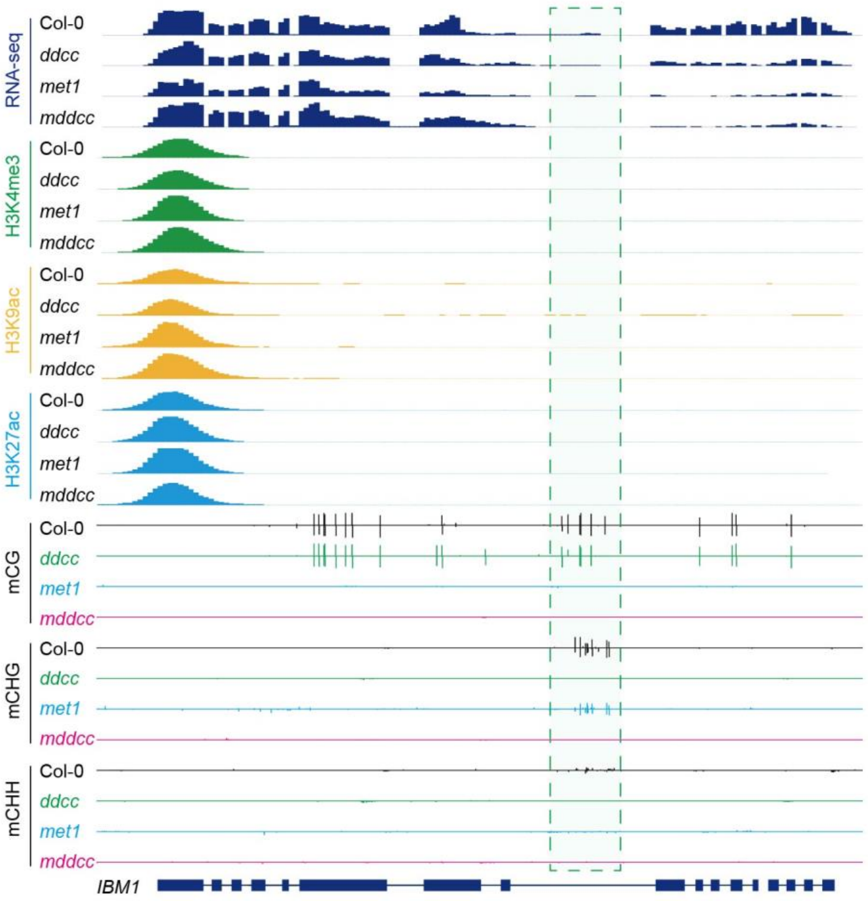

Fig. S9. DNA methylation is required for proper *IBM1* expression.

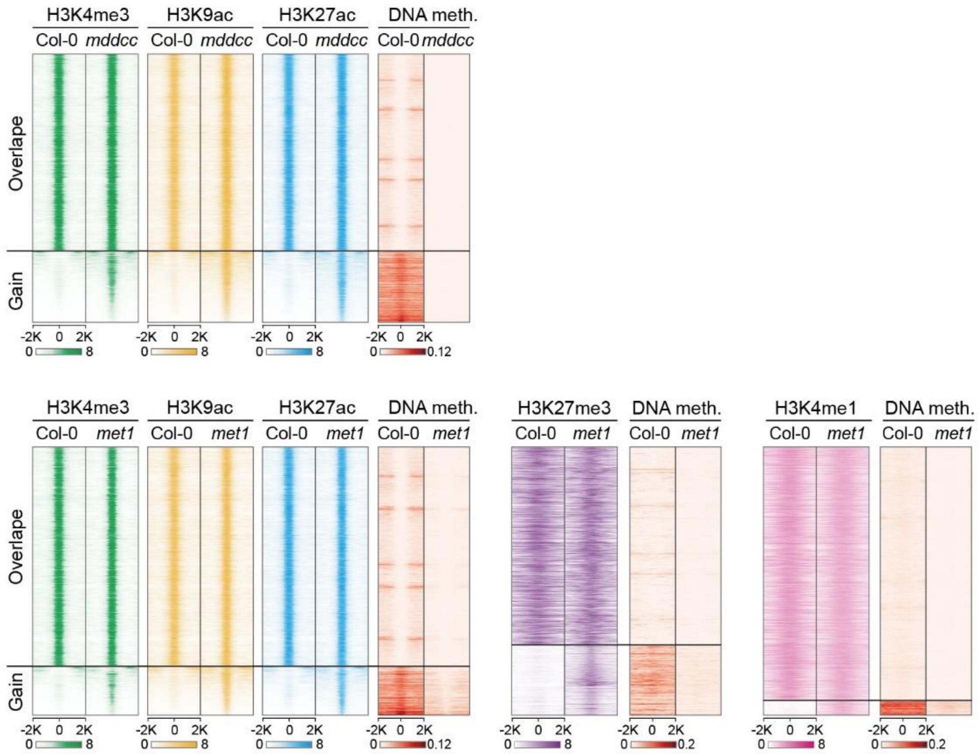

Fig. S10. Heatmap representation of histone marks and DNA methylation changes in the wild type and indicated mutants.

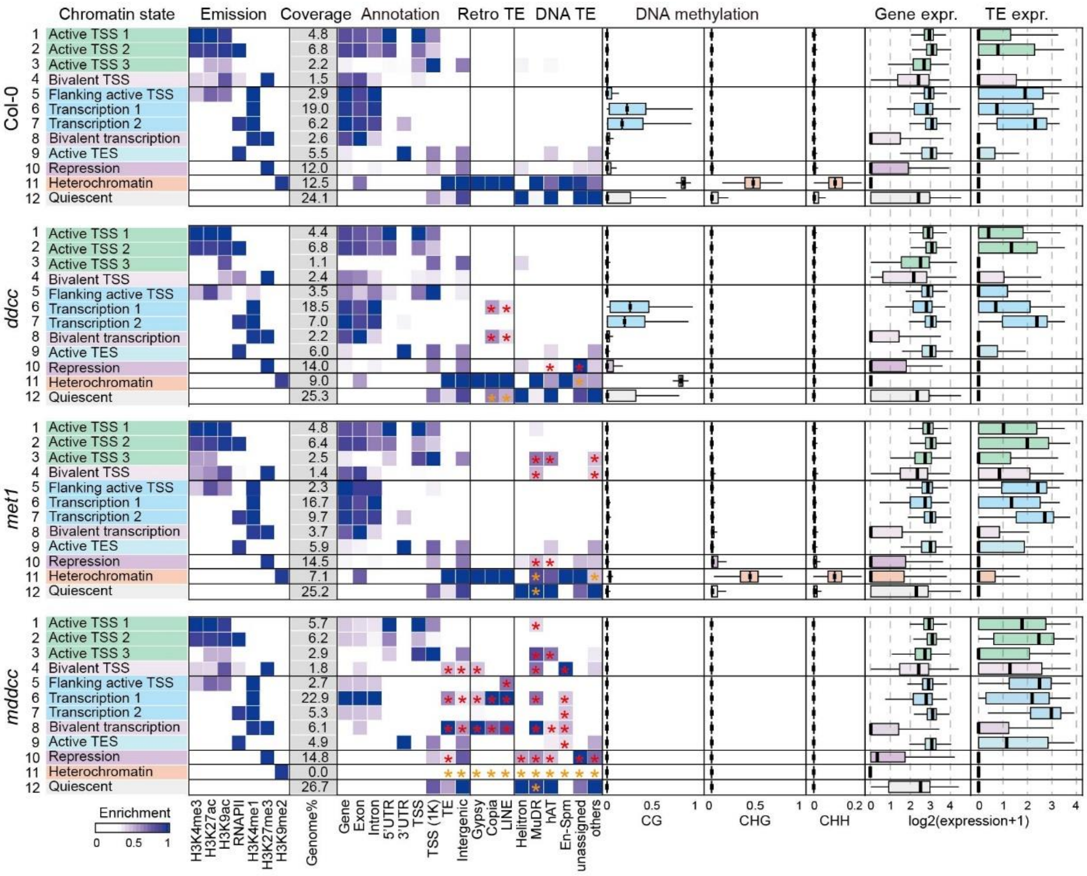

Fig. S11. Chromatin states, genomic annotation, TE enrichment, DNA methylation, and gene and TE expression in the indicated genotypes. Same/similar chromatin states are defined based on emission probability of six modified histones and RNAPII occupancy in all genotypes. Red and yellow asterisks indicate higher and lower enrichments in mutant than the wild type, respectively.

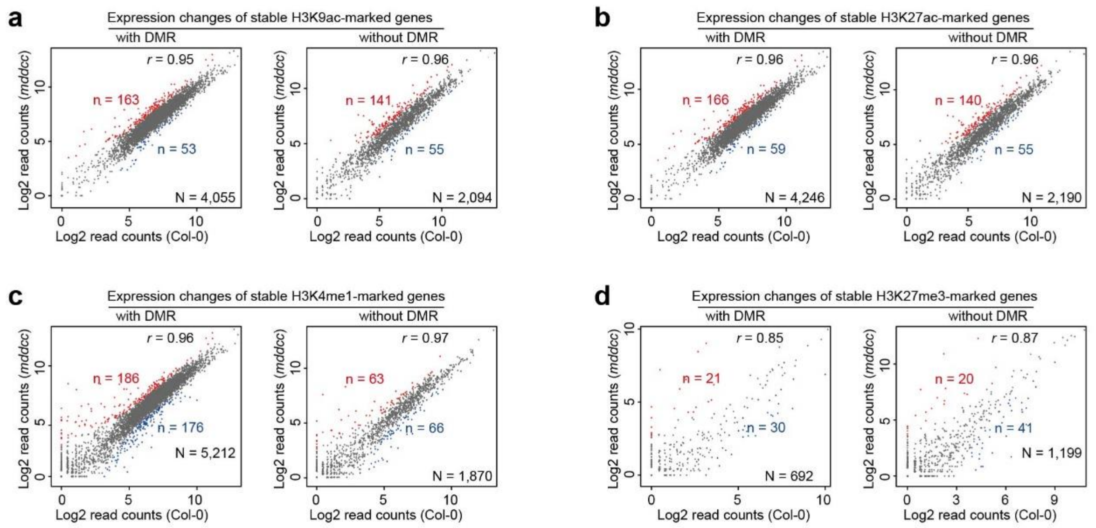

Fig. S12. Dot plot depicting the transcript changes in genes marked by stable histone marks associated with or not associated with differentially methylated regions (DMR) in *mddcc* and the wild type.

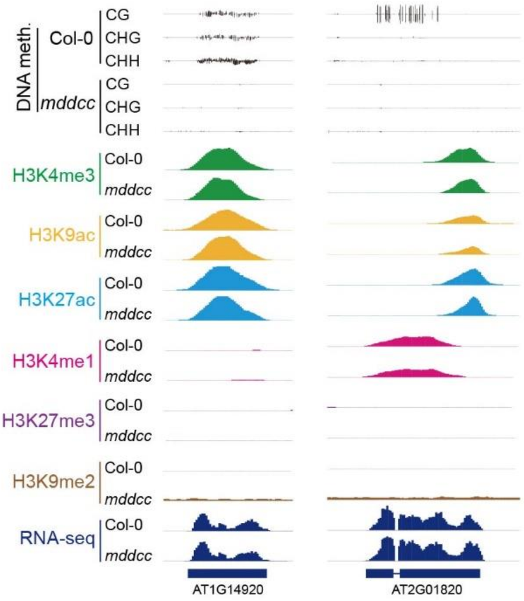

Fig. S13. Examples of no significant effect of DNA methylation on the expression of genes with stable histone modifications.

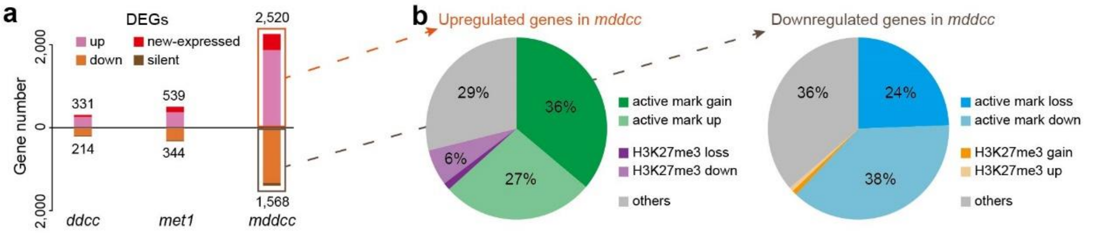

Fig. S14. Relationship between histone modifications and expression of protein-coding genes in DNA methylation-free mutants.

a, Numbers of differentially expressed genes (DEGs) in *ddcc*, *met1* and *mddcc* compared with the wild type. **b,** Histone modification compositions of DEGs in *mddcc*.

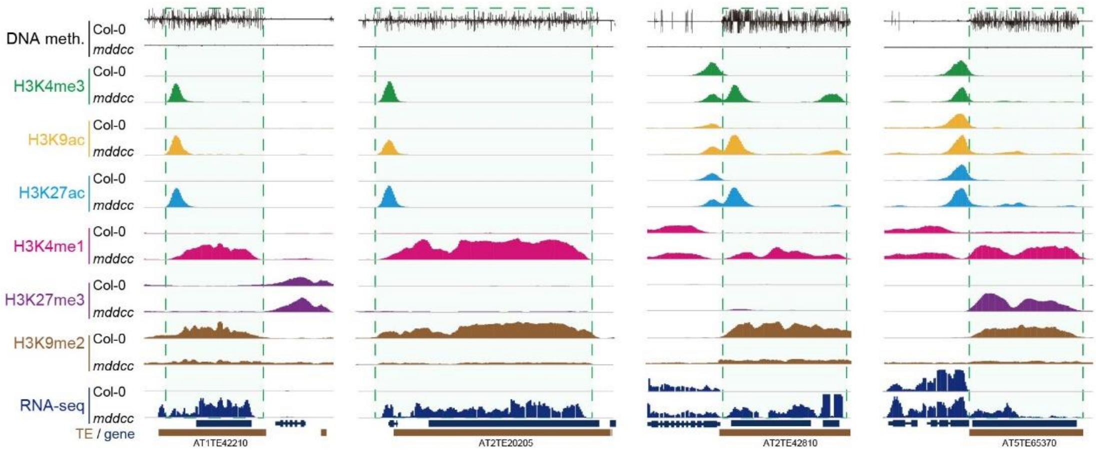

Fig. S15. The epigenetic patterns of transposed TEs in *mddcc*.
