# Supplementary material for: DNA methylation underpins the epigenomic landscape regulating genome transcription in Arabidopsis
Source: Genome Biol. 2022 Sep 20;23:197. doi: 10.1186/s13059-022-02768-x (PMC9487137; doi:10.1186/s13059-022-02768-x)
Supplement: Supplementary file 4 — Additional file 4: Review history. [file 13059_2022_2768_MOESM4_ESM.docx]

Review History

**First round of review**

**Reviewer 1**

**Were you able to assess all statistics in the manuscript, including the appropriateness of statistical tests used?**

Yes, and I have assessed the statistics in my report.

**Comments to author:**

This manuscript is a follow-up to the same group's earlier publication of an Arabidopsis mutant lacking all known DNA methyltransferases (He et al, Nat Commun 2022).

The authors conclude that DNA methylation acts as a scaffold, or signal, for other epigenetic modifications. This is consistent with previous understanding of the role of DNA methylation in, for example, promoting repressive histone modifications (for example in Mathieu et al, Cell 2007). Although there are many ChIPs and analyses here, I do not find that the work produces substantial new insight into the relationship between DNA methylation, histone modifications, and transcription.

1. Technically the study appears well done, although more detail should be included in the methods about parameters for read mapping. If default settings were used, indicate as such. Also indicate the length of reads sequenced for each experiment. Additionally, it is standard practice to have 3 replicates for ChIP-Seq. I'm not advocating that the authors do that here, just a note for future work.

2. The DNA methylation profiling is redundant with the earlier Nat Commun paper, and it is not clear why it is performed again here.

3. The approach of comparing 5-week old shoots for mddcc to two week old shoots is appropriate, given the severe developmental delay of mddcc.

4. The research shows that loss of DNA methylation leads to complete loss of H3K9me2. This is expected based on previous work on met1 and ddcc, but has not been previously demonstrated. A convincing argument is made that remaining H3K9me2 peaks in mddcc are artifacts.

5. The conclusion that (line 174) that "the interplay between DNA methylation and H3K9me2 goes beyond the well-known feedback regulatory loop between CHG methylation and H3K9me2" is a bit of a stretch - the effects of met1 on H3K9me2 were already known. The conclusions here appear essentially to be the same.

6. Throughout the manuscript, correlation is often conflated with causation. For example, in discussing gene expression changes (paragraph starting on line 275) it is concluded that transcriptional reprogramming upon loss of DNA methylation mainly occurs through the redistribution of histone modifications. It is not surprising that genes expressed in one genotype and not the other are associated with differential levels of active histone marks. It does not mean that those marks were the cause of the change in gene expression.

7. The previous paper extensively discussed TE expression in mddcc and the other mutants. The current analysis again appears redundant (Fig. 7).

**Reviewer 2**

**Were you able to assess all statistics in the manuscript, including the appropriateness of statistical tests used?**

Yes, and I have assessed the statistics in my report.

**Comments to author:**

The manuscript entitled: « DNA methylation underpins the epigenomic landscape regulating genome transcription in Arabidopsis» by Zhao et al., reports the role of DNA methylation in the establishment of the proper epigenome landscape.

The author used a quintuple mutant of the DNA methyltransferase to measure the role of the DNA methylation context on transcription and on 6 representative histone modifications.

The experiments are well performed and the use of unique genetic resources allow deciphering the redistribution of epigenetic marks in absence of DNA methylation.

In addition, the comparisons of the epigenomic landscapes between different DNA methylation mutant affected in the 3 contexts provide interesting knowledge and allows drawing a model.

Minor comments

Given that reduced or absence of DNA methylation would lead to TE mobilization and likely neo insertions, I strongly suggest to discuss the putative emergence of structural variations (SVs) in mddcc or ddcc plants. These SVs (i.e new TE insertions) may influence gene expression and enrichment of particular histone marks. It likely contributes to the redistribution of several histone marks.

In addition, the role of small RNA for preventing TE insertions (i.e siRNA) and thus for genome integrity could be included in the discussion (one or 2 sentences) to give an overview of the parallel mechanisms that may also participate in genome immunity together with the nice results reported in this study.

**Reviewer 3**

**Were you able to assess all statistics in the manuscript, including the appropriateness of statistical tests used?**

No, I do not feel adequately qualified to assess the statistics.

**Comments to author:**

Zhao et al. is a study that details the transcriptomic and chromatin mark changes within the recently described mddcc mutant that lacks all DNA methylation, in both the CG and non-CG contexts. This work is extensive, taking advantage of enhanced ChIP-seq (of multiple histone marks and RNAPII), RNA-seq and BS-seq and by combining this multiomics approach, the authors were able to discover new relationships between DNA methylation and genome regulation. Overall I found this work to be thorough and a pleasure to read. Its insights into the relationship of different histone marks with CG methylation, non-CG methylation or both types of methylation, and how this affects transcription is very important. This work's conclusions are well supported by their data (except where discussed) and is of broad interest to biologists with an interest in gene/TE regulation and epigenetics. Below are my comments on what changes I think could or should be made to improve the quality of the manuscript.

Intro:

Page 3 Lines 50-51: When discussing the interdependency of different DNA contexts, I think that you should cite the work Watson et al. (2014) https://journals.plos.org/plosone/article?id=10.1371/journal.pone.0105338

Page 3 Lines 61-64: When discussing which enzyme is responsible for which type of DNA methylation, I think that it would be worth mentioning the role of CMT2 in mCHH via H3K9me2, as found by Stroud et al. (2014) https://www.nature.com/articles/nsmb.2735

Results:

Page 5 Lines 97-98: I wonder if the term constitutive heterochromatin to describe the regions made inactive by H3K9me2 might be a good term/concept to introduce here and use throughout the manuscript. This going along with facultative heterochromatin to describe the regions repressed by H3K27me3, to bring it in line with how others describe this in the literature.

Page 5 Line 113: "Loss" not "Lost"

Page 5 Line 114: Perhaps add "DNA" between "heavily methylated" to add clarity to what is being methylated.

The data in Figure 2a could perhaps be better presented in a UpSet plot, rather than a multi-way Venn diagram.

Page 6 Lines 127-128: Can the authors please detail why this result is "consistent" with the finding that they are using it to support (I think I understand but many readers might not immediately appreciate the connection here).

Page 8 and onwards: At multiple points from here onwards, starting in a sub-heading title (line 176) and again in the text below (such as line 178), the authors repeat that plants gain repressive histone marks. This is confusing as the previous section did a thorough job of demonstrating that the repressive histone mark H3K9me2 was removed from the genome when the DNA methylation was removed. The only other repressive mark examined in this study was the Polycomb repressive complex related H3K27me3, which is responsible for facultative heterochromatin. Here the authors group some active marks together, which I think is fair, and the seperate out H3K4me1 as it does not fit into this category perfectly. So I think that the authors should not use the term "repressive" when what they really mean is H3K27me3. Be specific, especially when you are examining another repressive histone mark (K9me2) which does not follow the described pattern here. Describing it as facultative heterochromatin or simply as H3K27me3 would be an improvement over "repressive" and will decrease confusion to the reader.

Page 8 Lines 182-194: Please briefly describe what you mean by simulation in this context.

Page 8 Lines 190-191: The authors described how some loci have H3K27me3 in the endosperm but DNA methylation in the vegetative tissues. I would not use the word "replaced" here as that might lead people to think that endosperm becomes vegetative tissue, which it does not. Instead I would describe it as a difference between the two tissue types. Also, I think that it would be interesting, is possible, to compare the sites with H3K27me3 found by the cited study (37) overlap with the loci described in this study that gain H3K27m3 after loss of all DNA methylation?

Page 10 Line 231 and in multiple figure panels: the term overlapping or overlap is sometimes used to describe histone states at loci shared between the Wild Type plants and the mddcc mutant. I feel like this term, especially in the plots, is not very meaningful. While accurate in how the list of sites was generated (and overlap from the analysis of the two genotypes). Instead, I think a better term should be used, such as "consistent marks" or "stable marks" to make it clear that these are the set of histone marks that do not change in response to the loss of DNA methylation. Variable is used to describe those that do change when methylation is lost, and I think this term is fine. A more descriptive alternative could be "DNA methylation-sensitive marks".

Page 12 Line 283: I do not think that "Vice versa" is the best way to start this point. Perhaps a better way to get the reading thinking in the correct terms would be that "When we examined this from a chromatin centric view, we see that..."

Page 12 Line 285-289: I think that it is important at this point (and in the Discussion) to mention that not all Histone marks were examined. The key marks known to have substantial changes on the transcriptome were examined, but some marks, such as the repressive H4K20 methylation, were not. This is important as when the authors find some regions of the genome that lose DNA methylation but do not change chromatin marks or transcriptional activity, we cannot conclude that no other mark is replacing a repressive role of DNA methylation at these sites. DNA could be passive at this sites and have no function (which I think might be true at some or even many sites) but this cannot be concluded from this set of experiments and the authors should discuss this point.

Page 13 Lines 312-314: I would re-arrange the ordering of this sentence to place the "except for the loss of DNA methylation in mddcc" until after the sentence states "TE showed epigenetic patters similar to those in wild type" as I think that this would flow better and be easier for the reader to parse.

Page 14 Line 322: I would replace "re-repressed" with "now repressed". Also, regarding this point, I think some discussion somewhere about what might guides H3K27me3 to these sites that were previously methylated by DNA would be interesting.

Discussion:

I think that Supp Fig 15 should be moved to the main figures if possible (unless space limitations from the journal prevent this). I think that it is a very nice summary of the results and should be more readily accessible to the reader.

Page 16 Lines 367-370: "in other cases, TEs with heavy DNA methylation, regardless of H3K9me2 deposition, remain silent in the DNA methylation mutants even though there is no repressive mark (Supplementary Fig. 15b, case 3 and 4), indicating that DNA methylation and/or inactive marks are not required for TE silencing in these genomic regions" as I mentioned above, I think that this conclusion is too strong as the presence/absence of other repressive histone marks has not been assessed and this should be edited to reflect that.

Methods:

Page 17: Please detail the exact type of tissues collected - was it just leaves (rosette leaves only)? This is especially important given the two different growth stages examined.

Please indicate the length of the raw reads for each sequencing experiment as they come off the sequencer and before trimming.

It is great to see that the raw reads are published but it would be very helpful to share some of the processed data files so that others can analyse your work and see better how each step was performed, to make the work more reproducible. Using a free online data repository that generates a DOI (like Zenodo) I think would be a good place for this and then share that in the methods section. Processed data files already generated related to methylation or RNA-seq or histone mark peaks would be appropriate, along with any custom scripts used to analyse them.

Figures:

Fig 3b has a label of "Overlape" when I think it should be "overlap" but as I mentioned above, I think it would be better to give this a more descriptive name.

Fig 5b: I very much like this figure (and Fig 5a) and I think they are very important. But in Fig 5b I think that the use of the word "only" at the end of the label is not clear. Perhaps the label name should start with "only in met1" rather than "in met1 only" so it is more distinguishable from the labels that are not "only in". Perhaps the word only could even be in bold to make it very clear.

Fig 6b: What are the units of expression? Log2(expression+1) does not indicate the units that are being transformed here. Is it TPM or FPKM or something else? Same in Fig 7b and h.

**Authors Response**

**Point-by-point responses to the reviewers’ comments:**

*We sincerely thank the reviewers for their careful review of our manuscript and valuable comments. We have revised our manuscript according to their comments which have significantly improved our manuscript. Our point-to-point responses to all comments are as follows.*

Reviewer #1:

This manuscript is a follow-up to the same group's earlier publication of an Arabidopsis mutant lacking all known DNA methyltransferases (He et al, Nat Commun 2022).

The authors conclude that DNA methylation acts as a scaffold, or signal, for other epigenetic modifications. This is consistent with previous understanding of the role of DNA methylation in, for example, promoting repressive histone modifications (for example in Mathieu et al, Cell 2007). Although there are many ChIPs and analyses here, I do not find that the work produces substantial new insight into the relationship between DNA methylation, histone modifications, and transcription.

*Response: We thank the reviewer for the comments. This study is the latest advances based on our earlier work (He et al, Nat Commun, 2022). The main results and conclusions of these two studies are different. In our Nat Commun paper, we have generated the DNA methylation-free Arabidopsis mutant mddcc for the first time, and revealed the crucial roles of DNA methylation in regulating genome transcription and development. In this study, we took advantage of the eChIP-seq method and mddcc material together with the met1 and ddcc mutants to investigate the roles of DNA methylation in the establishment of genome-wide histone modification landscape and in transcriptional control. In our opinion which is also supported by the other reviewers’ comments, some of the novel findings in our manuscript are as follows:*

*(1) We have generated a large amount and high-quality epigenome data (over 120 data sets) for Arabidopsis wild type and mutants that are devoid of CG (met1) methylation, non-CG (ddcc) methylation, or total DNA methylation (mddcc), that will serve as a valuable resource to the research community for further studies.*

*(2) We evaluated in a more quantitative manner the contribution of CG and non-CG DNA methylation in the deposition of heterochromatin mark H3K9me2. In contrast to the widely known notion that H3K9me2 mainly depends on non-CG methylation alone (Stroud et al, Nat Struct Mol Biol 2014), 1) we demonstrated for the first time that the complete loss of H3K9me2 occurs in the totally DNA methylation-free mutant mddcc; 2) CG methylation (specifically regulating ~30% of total H3K9me2 loci) has a greater effect on H3K9me2 deposition than non-CG methylation (~11%); 3) CG and non-CG methylation can act independently at some genomic regions while they act cooperatively at many other regions. Please see our response to point #5 of your comments for more explanation on this issue.*

*(3) We also determined the relative contributions of CG and non-CG methylation in the upheaval of the epigenetic landscape and transcription, and found that total loss of DNA methylation triggered transcriptional reprogramming correlates with the extensive redistribution and switches of histone modifications.*

*(4) Using the DNA methylation-free Arabidopsis mutant plants, we discovered for the first time in plants that the active histone marks (H3K4me3 and H3K9ac) and the repressive mark H3K27me3 can serve as DNA methylation-independent transcriptional regulatory systems.*

*(5) Our observation that H3K27me3 compensates for the loss of heavy DNA methylation/H3K9me2 in transposon silencing supports the concept that plants have evolved multiple systems to ensure genome stability.*

*Collectively, our results not only provide an invaluable resource for the epigenetics research community but also yield new insights into the contribution of DNA methylation to the plasticity of the epigenomic landscape as well as the functions of this complex epigenetic regulatory network in genome transcription. While our findings are, not surprisingly, overall consistent with previous understanding of the role DNA methylation in shaping the histone modification landscape, which was based on many excellent studies, our findings clearly add to, extend and complement previous work and improves our understanding.*

1. Technically the study appears well done, although more detail should be included in the methods about parameters for read mapping. If default settings were used, indicate as such. Also indicate the length of reads sequenced for each experiment. Additionally, it is standard practice to have 3 replicates for ChIP-Seq. I'm not advocating that the authors do that here, just a note for future work.

*Response: We appreciate the reviewer’s positive comments and suggestions. We have added the detailed parameters of the software and marked the length of the sequencing data. We have revised these throughout the manuscript as follows:*

***ChIP-seq and data analysis.***

*Libraries were prepared using an NEBNext® Ultra™ II DNA library prep kit for Illumina® sequencing (New England BioLabs, E7645) and sequenced using an Illumina HiSeq X Ten platform (paired-end 150-bp reads).*

*Raw reads were first trimmed as paired-end reads using Fastp with default parameters. Clean reads were mapped to the TAIR10 Arabidopsis genome by BWAMEM with default parameters.*

***Whole-genome bisulfite DNA sequencing (BS-seq) and data analysis.***

*The libraries were then sequenced using an Illumina HiSeq X Ten platform (paired-end 150-bp reads). For data analysis, low-quality read trimming and artificial sequence trimming were performed using Fastp with default parameters. Clean reads were mapped to the Arabidopsis genome (TAIR10) using BatMeth2-align with default parameters. DNA methylation calling was performed with BatMeth2-calmeth with parameter ‘-n 0.1 -Q 30’, and the SAM file was converted to the BAM format with SAMtools. We then used batmeth2_to_bigwig.py scripts to generate BigWig files for visualization using IGV.*

**Total RNA-seq and data analysis.**

*Raw reads were first trimmed as paired-end reads using Fastp with default parameters to remove the adaptors and low-quality reads. Clean reads were mapped to the Arabidopsis genome (TAIR10) using Hisat2 with default parameters, and then SAMtools was used to sort the BAM file and remove the low map quality reads with - q 30.*

*We also appreciate your advice about the standard of ChIP-seq replicates.*

2. The DNA methylation profiling is redundant with the earlier Nat Commun paper, and it is not clear why it is performed again here.

*Response: Thank you for the comment. The DNA methylation profiling is not the main content and novel finding of this study. Both two- and five-week-old WT, met1 and ddcc plants were used in this study. Thus, we presented DNA methylation profiling for two- and five-week-old WT, met1, and ddcc in our earlier study (He et al, Nat Commun 2022) and this study (Supplementary Fig. 1), respectively, to confirm the correctness of these materials used in this study. Considering the integrity of this study and the convenience for readers to understand, we have revised these panels and descriptions as basic or supporting conclusion for key findings.*

*Supplementary Figure 1. Dissecting contributions of DNA methyltransferases to DNA methylation patterns in five-week-old plants.*

*Genome browser view and average distribution of CG, CHG, and CHH methylation over TEs and genes in five-week-old plants of the indicated genotypes. Flanking regions are 2 kb upstream and downstream of the gene or TE body. TSS, transcription start site. TTS, transcription termination site.*

3. The approach of comparing 5-week old shoots for mddcc to two week old shoots is appropriate, given the severe developmental delay of mddcc.

*Response: Thank you for the comment.*

4. The research shows that loss of DNA methylation leads to complete loss of H3K9me2. This is expected based on previous work on met1 and ddcc, but has not been previously demonstrated. A convincing argument is made that remaining H3K9me2 peaks in mddcc are artifacts.

*Response: Thank you for the comment.*

5. The conclusion that (line 174) that "the interplay between DNA methylation and H3K9me2 goes beyond the well-known feedback regulatory loop between CHG methylation and H3K9me2" is a bit of a stretch - the effects of met1 on H3K9me2 were already known. The conclusions here appear essentially to be the same.

*Response: Thank you for the comment. Yes, the feedback regulatory loop between CHG methylation and H3K9me2 is well-known (reviewed by Du et al. Nat Rev Mol Cell Biol 2015), and previous studies reported that elimination of CG methylation in met1 mutant results in loss of H3K9me2 (Mathieu et al, Cell 2007; Stroud et al, Nat Struct Mol Biol 2014). Of note, the Mathieu et al Cell paper relied on immunostaining approach to study the effect of DNA methylation on H3K9me2 deposition in a qualitative rather than quantitative manner. Stroud et al (2014) and Du et al (2015) reported that elimination of all non-CG methylation in ddcc mutant results in loss of total (or almost all) H3K9me2, whereas elimination of CG methylation in met1 mutant caused the loss of a limited number of H3K9me2 loci, which suggested that H3K9me2 is much more dependent on non-CG methylation than CG methylation.*

*In this study, we took advantage of the eChIP method and the DNA methylationfree mutant mddcc together with met1 (CG methylation-free mutant) and ddcc (nonCG methylation-free mutant) to generate high-quality H3K9me2 ChIP-seq data, and thus obtained some novel and/or different results from the previous studies. 1) As mentioned above, we demonstrated for the first time that that complete loss of H3K9me2 occurs in the totally DNA methylation-free mutant mddcc, in contrast to the notion that H3K9me2 mainly depends on non-CG methylation alone (Stroud et al, 2014). 2) We quantitatively examined the effects of CG and non-CG methylation on H3K9me2 loci: CG methylation (specifically regulating ~30% of total H3K9me2 loci) has a greater effect on H3K9me2 deposition than non-CG methylation (~11%). 3) In addition, DNA methylation-related mutants, especially DNA methylation-free mutant mddcc, are the basis for comprehensively evaluating the contribution of CG and nonCG methylation to H3K9me2 deposition: CG and non-CG methylation can act independently at some genomic regions while they act cooperatively at many other regions. 4) Finally, as shown in Figure 8, we proposed distinct models to illustrate the complex functional links between DNA methylation and H3K9me2 based on our work and the work of others.*

6. Throughout the manuscript, correlation is often conflated with causation. For example, in discussing gene expression changes (paragraph starting on line 275) it is concluded that transcriptional reprogramming upon loss of DNA methylation mainly occurs through the redistribution of histone modifications. It is not surprising that genes expressed in one genotype and not the other are associated with differential levels of active histone marks. It does not mean that those marks were the cause of the change in gene expression.

*Response: Thank you for the comment. We have revised these sentences as follows: “Most of these expression changes correlated with the corresponding changes in histone modifications:” (Lines 285-286 in the revised manuscript). “The transcriptional reprogramming upon loss of all DNA methylation correlates with the extensive redistribution or switches of the examined histone modifications.” (Lines 35-37); “we found that the expression level of most genes with gain/increase of active marks were up-regulated, whereas these with loss/decrease of active marks were down-regulated (Fig. 6e).” (Lines 290-292); “the transcriptional reprogramming observed upon loss of DNA methylation corelates with the redistribution of the examined histone modifications or through the modulation of the levels of histone modifications.” (Lines 293-296); “Our results demonstrate that transcriptional reprogramming resulting from the loss of DNA methylation is largely associated with the redistribution of the examined histone modifications and switches of chromatin states.” (Lines 372-374).*

7. The previous paper extensively discussed TE expression in mddcc and the other mutants. The current analysis again appears redundant (Fig. 7).

*Response: Thank you for the comment. Yes, both studies analyzed TE expression and obtained similar results. In this study, we also investigated TE expression and features in the wild type. The analysis of TE expression served as the basis for further analysis of the relationship between DNA methylation, histone modifications and TE activity. Therefore, considering the integrity of this study and the convenience for readers to understand, we present this panel as basic or supporting conclusion for key findings.*

Reviewer #2:

The manuscript entitled: « DNA methylation underpins the epigenomic landscape regulating genome transcription in Arabidopsis» by Zhao et al., reports the role of DNA methylation in the establishment of the proper epigenome landscape.

The author used a quintuple mutant of the DNA methyltransferase to measure the role of the DNA methylation context on transcription and on 6 representative histone modifications.

The experiments are well performed and the use of unique genetic resources allow deciphering the redistribution of epigenetic marks in absence of DNA methylation.

In addition, the comparisons of the epigenomic landscapes between different DNA methylation mutant affected in the 3 contexts provide interesting knowledge and allows drawing a model.

*Response: Thank you for the positive comments.*

Minor comments

Given that reduced or absence of DNA methylation would lead to TE mobilization and likely neo insertions, I strongly suggest to discuss the putative emergence of structural variations (SVs) in mddcc or ddcc plants. These SVs (i.e new TE insertions) may influence gene expression and enrichment of particular histone marks. It likely contributes to the redistribution of several histone marks.

In addition, the role of small RNA for preventing TE insertions (i.e siRNA) and thus for genome integrity could be included in the discussion (one or 2 sentences) to give an overview of the parallel mechanisms that may also participate in genome immunity together with the nice results reported in this study.

*Response: Thank you for this suggestion. As our earlier report, CG and non-CG methylation act redundantly to prevent TEs transposition (He, et al. Nat Commun. 2022). By analyzing the DNA-seq data and PCR confirmation, we identified 0, 0, and only 10 TE transposition events in ddcc, met1, and mddcc, respectively. Thus, loss of DNA methylation resulted in a small number of TE transposition events, which may also be inhibited by other uncovered regulatory mechanisms in Arabidopsis.*

*Following the reviewer’s advice, we added discussion about the role of DNA methylation in TE transposition and the redistribution of histone marks as follows: “we identified 10 TE transposition events involving 4 TEs in mddcc in our previous study [28]. We then examined the chromatin states of these 4 TEs and found that all of these TEs previously repressed by heavy DNA methylation and H3K9me2 in the wild type were marked by active marks and showed high levels of expression upon loss of total DNA methylation (Supplementary Fig. 15)” (Lines 321-325). “All TE transposition events identified in mddcc [28] belong to the first category (Fig. 8b, case 1), suggesting that chromatin state transition switches, especially between inactive and active states, may participate in TE transposition and genome integrity in mammals and plants.” (Lines 379-381).*

*Supplementary Figure 15. The epigenetic patterns of transposed TEs in mddcc.*

Reviewer #3:

Zhao et al. is a study that details the transcriptomic and chromatin mark changes within the recently described mddcc mutant that lacks all DNA methylation, in both the CG and non-CG contexts. This work is extensive, taking advantage of enhanced ChIP-seq (of multiple histone marks and RNAPII), RNA-seq and BS-seq and by combining this multiomics approach, the authors were able to discover new relationships between DNA methylation and genome regulation. Overall I found this work to be thorough and a pleasure to read. Its insights into the relationship of different histone marks with CG methylation, non-CG methylation or both types of methylation, and how this affects transcription is very important. This work's conclusions are well supported by their data (except where discussed) and is of broad interest to biologists with an interest in gene/TE regulation and epigenetics. Below are my comments on what changes I think could or should be made to improve the quality of the manuscript.

*Response: We really appreciate your positive comments and the careful review of our manuscript.*

Intro:

Page 3 Lines 50-51: When discussing the interdependency of different DNA contexts, I think that you should cite the work Watson et al. (2014) <https://journals.plos.org/plosone/article?id=10.1371/journal.pone.0105338>.

*Response: Following the reviewer’s suggestion, we have cited the work of Watson et al. (2014).*

Page 3 Lines 61-64: When discussing which enzyme is responsible for which type of DNA methylation, I think that it would be worth mentioning the role of CMT2 in mCHH via H3K9me2, as found by Stroud et al. (2014) <https://www.nature.com/articles/nsmb.2735>

*Response: Following the reviewer’s advice, here we have cited the work of Stroud et al. (2014) and revised this sentence as below: “Non-CG methylation and H3K9me2 reinforce each other through a feedback loop: methylated CHG and CHH recruit the H3K9-specific methyltransferases SUVH4, SUVH5, and SUVH6, and in turn, H3K9me2 facilitates CMT3 and CMT2 function to methylate DNA in the CHG and/or CHH context [8, 18-20].”*

Results:

Page 5 Lines 97-98: I wonder if the term constitutive heterochromatin to describe the regions made inactive by H3K9me2 might be a good term/concept to introduce here and use throughout the manuscript. This going along with facultative heterochromatin to describe the regions repressed by H3K27me3, to bring it in line with how others describe this in the literature.

*Response: We agree. Following your suggestions and the literature (i.e. Stefano, et al. Nucleic Acids Research. 2021; Zhang, et al. Genome Research. 2021; Montgomery, et al. Current Biology. 2020; Deleris, et al. PLOS Genetics. 2012), we have changed the sentence as suggested “H3K27me3 is deposited by Polycomb group proteins and is associated with facultative heterochromatin; and H3K9me2 is associated with constitutive heterochromatin.”, and have checked the use of H3K9me2 and H3K27me3 throughout the manuscript.*

Page 5 Line 113: "Loss" not "Lost"

*Response: We have changed this word as suggested.*

Page 5 Line 114: Perhaps add "DNA" between "heavily methylated" to add clarity to what is being methylated.

*Response: Thank you for the comment. We have added "DNA" between "heavily methylated".*

The data in Figure 2a could perhaps be better presented in a UpSet plot, rather than a multi-way Venn diagram.

*Response: Thank you for the comment. We have revised this figure as suggested.*

*Figure 2a, Upset plot displaying the number of unique and overlapping H3K9me2 loci between the wild type and indicated mutants. Bars represent the intersection size for overlapping H3K9me2 loci between Col-0, met1, ddcc, and mddcc. Numbers (>10) of H3K9me2 loci in different subgroups are shown. All H3K9me2 loci were classified into eight types as indicated.*

Page 6 Lines 127-128: Can the authors please detail why this result is "consistent" with the finding that they are using it to support (I think I understand but many readers might not immediately appreciate the connection here).

*Response: Thank you for the comment. To make it clearer, we revised this sentence as “high transcript levels were detected at these H3K9me2 loci in mddcc, indicating that these loci are active genomic regions rather than inactive/heterochromatic regions”.*

Page 8 and onwards: At multiple points from here onwards, starting in a sub-heading title (line 176) and again in the text below (such as line 178), the authors repeat that plants gain repressive histone marks. This is confusing as the previous section did a thorough job of demonstrating that the repressive histone mark H3K9me2 was removed from the genome when the DNA methylation was removed. The only other repressive mark examined in this study was the Polycomb repressive complex related H3K27me3, which is responsible for facultative heterochromatin. Here the authors group some active marks together, which I think is fair, and the seperate out H3K4me1 as it does not fit into this category perfectly. So I think that the authors should not use the term "repressive" when what they really mean is H3K27me3. Be specific, especially when you are examining another repressive histone mark (K9me2) which does not follow the described pattern here. Describing it as facultative heterochromatin or simply as H3K27me3 would be an improvement over "repressive" and will decrease confusion to the reader.

*Response: Thank you for this suggestion. We have replaced “repressive mark” with “H3K27me3” and replaced “inactive” with “H3K9me2” throughout the manuscript to make it more accurate.*

Page 8 Lines 182-194: Please briefly describe what you mean by simulation in this context.

*Response: The description of simulation was added to the figure legend of Figure 3a in the revised manuscript as follows: “In our simulation, un-modified regions were uniformly sampled as simulated peaks, and the lengths of the simulated peaks follow the similar length distribution of the real peaks.”*

Page 8 Lines 190-191: The authors described how some loci have H3K27me3 in the endosperm but DNA methylation in the vegetative tissues. I would not use the word "replaced" here as that might lead people to think that endosperm becomes vegetative tissue, which it does not. Instead I would describe it as a difference between the two tissue types. Also, I think that it would be interesting, is possible, to compare the sites with H3K27me3 found by the cited study (37) overlap with the loci described in this study that gain H3K27m3 after loss of all DNA methylation?

*Response: Thank you for this suggestion. Following your advice, this sentence has been revised as “Previous studies found that some genomic loci were specifically modified by H3K27me3 in the endosperm, whereas these loci were marked by DNA methylation in vegetative tissues.” In addition, we obtained 206 gain- H3K27me3 gene in the endosperm from the cited paper (Weinhofer et al, 2010), and analyzed the overlap between these genes and gain-H3K27me3 genes in mddcc. The overlap result is shown below:*

Please indicate the length of the raw reads for each sequencing experiment as they come off the sequencer and before trimming.

*Response: We have indicated the length of raw reads in the manuscript as follows:*

***ChIP-seq and data analysis.***

*Libraries were prepared using an NEBNext® Ultra™ II DNA library prep kit for*

*Illumina® sequencing (New England BioLabs, E7645) and sequenced using an Illumina*

*HiSeq X Ten platform (paired-end 150-bp reads).*

***Whole-genome bisulfite DNA sequencing (BS-seq) and data analysis.***

*The libraries were then sequenced using an Illumina HiSeq X Ten platform (paired-end 150-bp reads).*

***Total RNA-seq and data analysis.***

*Total RNA was isolated using the RNeasy Plant Mini Kit (QIAGEN, 74904); library preparation was performed at the PSC Genomics Core Facility according to the manufacturer’s instructions (Illumina). The libraries were sequenced on an Illumina HiSeq X Ten platform (paired-end 150-bp reads).*

Page 10 Line 231 and in multiple figure panels: the term overlapping or overlap is sometimes used to describe histone states at loci shared between the Wild Type plants and the mddcc mutant. I feel like this term, especially in the plots, is not very meaningful. While accurate in how the list of sites was generated (and overlap from the analysis of the two genotypes). Instead, I think a better term should be used, such as "consistent marks" or "stable marks" to make it clear that these are the set of histone marks that do not change in response to the loss of DNA methylation. Variable is used to describe those that do change when methylation is lost, and I think this term is fine. A more descriptive alternative could be "DNA methylation-sensitive marks".

*Response: Thank you for this suggestion. Following your advice, we have replaced the term “overlapping or overlap” to “stable mark” and used “variable” to describe these changed epigenetic marks throughout the manuscript including the figure panels and legends.*

Page 12 Line 283: I do not think that "Vice versa" is the best way to start this point. Perhaps a better way to get the reading thinking in the correct terms would be that "When we examined this from a chromatin centric view, we see that..."

*Response: Thank you for this suggestion. We have revised this sentence as suggested.*

Page 12 Line 285-289: I think that it is important at this point (and in the Discussion) to mention that not all Histone marks were examined. The key marks known to have substantial changes on the transcriptome were examined, but some marks, such as the repressive H4K20 methylation, were not. This is important as when the authors find some regions of the genome that lose DNA methylation but do not change chromatin marks or transcriptional activity, we cannot conclude that no other mark is replacing a repressive role of DNA methylation at these sites. DNA could be passive at this sites and have no function (which I think might be true at some or even many sites) but this cannot be concluded from this set of experiments and the authors should discuss this point.

*Response: Thank you for this suggestion. We have revised these sentences as “The transcriptional reprogramming upon loss of all DNA methylation correlates with the extensive redistribution or switches of the examined histone modifications.” (Lines 35- 37); “the transcriptional reprogramming observed upon loss of DNA methylation correlates with the redistribution of the examined histone modifications or through the modulation of the levels of histone modifications.” (Lines 293-296); “Our results demonstrate that transcriptional reprogramming resulting from the loss of DNA methylation is largely associated with the redistribution of the examined histone modifications and switches of chromatin states.” (Lines 372-374).*

Page 13 Lines 312-314: I would re-arrange the ordering of this sentence to place the "except for the loss of DNA methylation in mddcc" until after the sentence states "TE showed epigenetic patters similar to those in wild type" as I think that this would flow better and be easier for the reader to parse.

*Response: Thank you for this suggestion. We have revised this sentence as suggested.*

Page 14 Line 322: I would replace "re-repressed" with "now repressed". Also, regarding this point, I think some discussion somewhere about what might guides H3K27me3 to these sites that were previously methylated by DNA would be interesting.

*Response: We appreciate your suggestions. We have replaced "re-repressed" with "now repressed". A brief discussion was added in Discussion section (Lines 399-404): “Ectopic gain of H3K27me3 in mddcc at some loci previously marked by DNA methylation and/or H3K9me2 (Fig. 4c and 7e) implies that DNA and H3K9 methyltransferases have stronger affinity than the H3K27me3 methyltransferases at these loci in the wild type. The chromatin environment lacking DNA methylation and H3K9me2 in the mddcc mutant probably facilitates the recruitment of Polycomb Repressive Complex 2 to deposit the H3K27me3 mark at these loci.”*

Discussion:

I think that Supp Fig 15 should be moved to the main figures if possible (unless space limitations from the journal prevent this). I think that it is a very nice summary of the results and should be more readily accessible to the reader.

*Response: Thank you for the suggestion. Following your suggestion, Supp Fig. 15 has been moved to the main figures (Fig. 8).*

Page 16 Lines 367-370: "in other cases, TEs with heavy DNA methylation, regardless of H3K9me2 deposition, remain silent in the DNA methylation mutants even though there is no repressive mark (Supplementary Fig. 15b, case 3 and 4), indicating that DNA methylation and/or inactive marks are not required for TE silencing in these genomic regions" as I mentioned above, I think that this conclusion is too strong as the presence/absence of other repressive histone marks has not been assessed and this should be edited to reflect that.

*Response: Thank you for the comment. Following the your suggestion, we have revised this sentence as “In other cases, TEs with heavy DNA methylation, regardless of H3K9me2 deposition, remain silent in the DNA methylation mutants even though there is no H3K27me3 mark (Fig. 8b, case 3 and 4), indicating that some undetected repressive marks and/or uncovered regulatory mechanisms may exist in these regions.”.*

Methods:

Page 17: Please detail the exact type of tissues collected - was it just leaves (rosette leaves only)? This is especially important given the two different growth stages examined.

*Response: Thank you for this suggestion. We have described the materials and tissues used in the study in detail: “The aerial part of two-week-old plants for the wild type, met1, and ddcc, and the aerial part (with the inflorescence removed) of five-week-old plants for the wild type, met1, ddcc, and mddcc were used for all experiments.”.*

Please indicate the length of the raw reads for each sequencing experiment as they come off the sequencer and before trimming.

*Response: The length of the raw reads is paired-end 150-bp. We have indicated this throughout the manuscript.*

It is great to see that the raw reads are published but it would be very helpful to share some of the processed data files so that others can analyse your work and see better how each step was performed, to make the work more reproducible. Using a free online data repository that generates a DOI (like Zenodo) I think would be a good place for this and then share that in the methods section. Processed data files already generated related to methylation or RNA-seq or histone mark peaks would be appropriate, along with any custom scripts used to analyse them.

*Response: Thank you for the comment. We sorted out the analysis process scripts of ChIP-seq, RNA-seq and WGBS data, as well as intermediate results such as BigWig, peak files, and gene expression files. The script and result files have been uploaded to Zenodo, with a total of 4.2G. The data link is as follow: https://doi.org/10.5281/zenodo.6575422. We have added the related description in the revised manuscript.*

Figures:

Fig 3b has a label of "Overlape" when I think it should be "overlap" but as I mentioned above, I think it would be better to give this a more descriptive name.

*Response: We have revised it as suggested.*

Fig 5b: I very much like this figure (and Fig 5a) and I think they are very important. But in Fig 5b I think that the use of the word "only" at the end of the label is not clear. Perhaps the label name should start with "only in met1" rather than "in met1 only" so it is more distinguishable from the labels that are not "only in". Perhaps the word only could even be in bold to make it very clear.

*Response: We have revised it as suggested.*

Fig 6b: What are the units of expression? Log2(expression+1) does not indicate the units that are being transformed here. Is it TPM or FPKM or something else? Same in Fig 7b and h.

*Response: We appreciate your comments and suggestions. To compare the gene expression between samples, we used TMM (Trimmed mean of M values standardization) standardization in edgeR for gene expression. As far as we know, TMM is superior to FPKM and TPM in comparisons between samples and is already used in RNA-seq differential gene analysis and gene expression visualization (Fernandez-Calero et al, Cancer Metab. 2020). In Zhao et al, J Transl Med 2021, this paper compared different standardized methods in detail, and gave the conclusion that DESeq2 normalized count data and TMM normalized count data generally performed better than the other quantification measures (ex. TPM, FPKM). In addition, we have added a corresponding note to the figure legend: Gene expression was standardized with TMM.*

**Second round of review**

**Reviewer 1**

The manuscript is improved, with more precise language and methodological details throughout. I appreciate the authors more clearly stating the contribution of their study to the literature.

Some instances remain where correlation and causation are conflated, for example:

(line 328-330) These results indicate that removal of DNA methylation and H3K9me2 provides a permissive state for TE derepression, but active marks are required for TE activation.

Rather than being 'required', active marks are correlated with TE expression. Indeed, it would be hard to find a Pol II-transcribed active region of the genome that lacks 'active' histone modifications. The authors should carefully check for such instances of conflating correlation and causation throughout.

**Reviewer 2**

The authors replied to all points and improved the manuscript.

**Reviewer 3**

Thank you for addressing my concerns. This is a wonderful study that I think is very important for the community.

Reviewer thinking to self: I wonder how long before someone knocks out the polycomb pathway in the mddcc mutant - I am sure that will also be very interesting (if viable). Any study that can make me wonder things like that is one I will remember.

**Authors Response**

**Point-by-point responses to the reviewers’ comments:**

Reviewer #1:

The manuscript is improved, with more precise language and methodological details throughout. I appreciate the authors more clearly stating the contribution of their study to the literature.

Some instances remain where correlation and causation are conflated, for example:

(line 328-330) These results indicate that removal of DNA methylation and H3K9me2 provides a permissive state for TE derepression, but active marks are required for TE activation.

Rather than being 'required', active marks are correlated with TE expression. Indeed, it would be hard to find a Pol II-transcribed active region of the genome that lacks 'active' histone modifications. The authors should carefully check for such instances of conflating correlation and causation throughout.

*Response: Thank you for the comment. Following the reviewer’s advice, we have revised this sentence as follow: These results indicate that removal of DNA methylation and H3K9me2 provides a permissive state for TE derepression, while ectopic gain of active marks is highly correlates with TE activation upon loss of DNA methylation. In addition, we have checked the use of correlation and causation throughout the revised manuscript.*

Reviewer #2:

The authors replied to all points and improved the manuscript.

*Response: Thank you for the comment.*

**Reviewer #3:**

Thank you for addressing my concerns. This is a wonderful study that I think is very important for the community.

Reviewer thinking to self: I wonder how long before someone knocks out the polycomb pathway in the mddcc mutant - I am sure that will also be very interesting (if viable). Any study that can make me wonder things like that is one I will remember.

*Response: I can’t agree you more. This is a very interesting and important issue for the epigenetics research community. Polycomb pathway-deposited H3K27me3 is critical for the control of transcription of many key genes and development. In this study, we spend a lot of time to collect enough mddcc materials which grew extremely slowly and failed to flower. If Arabidopsis mutant completely devoid of H3K27me3 is not embryonic lethal, it is possible to obtain a mutant completely devoid of both DNA methylation and H3K27me3. Together, although it is theoretically possible to remove all DNA methylation and H3K27me3 simultaneously in Arabidopsis, it should be a very difficult process. However, it is still a thing worth trying boldly.*
